# Gene expression signatures in motor neurone disease fibroblasts reveal dysregulation of metabolism, hypoxia-response and RNA processing functions

**DOI:** 10.1111/nan.12147

**Published:** 2015-01-29

**Authors:** R Raman, S P Allen, E F Goodall, S Kramer, L-L Ponger, P R Heath, M Milo, H C Hollinger, T Walsh, J R Highley, S Olpin, C J McDermott, P J Shaw, J Kirby

**Affiliations:** * Sheffield Institute for Translational Neuroscience (SITraN), Department of Neuroscience, University of SheffieldSheffield, UK; † Department of Biomedical Science, University of SheffieldSheffield, UK; ‡ Metabolic Biochemistry, Sheffield Children's NHS Foundation TrustSheffield, UK

**Keywords:** amyotrophic lateral sclerosis, cell models, fibroblasts, hypoxia response, microarray, microRNA, primary lateral sclerosis

## Abstract

**Aims:**

Amyotrophic lateral sclerosis (ALS) and primary lateral sclerosis (PLS) are two syndromic variants within the motor neurone disease spectrum. As PLS and most ALS cases are sporadic (SALS), this limits the availability of cellular models for investigating pathogenic mechanisms and therapeutic targets. The aim of this study was to use gene expression profiling to evaluate fibroblasts as cellular models for SALS and PLS, to establish whether dysregulated biological processes recapitulate those seen in the central nervous system and to elucidate pathways that distinguish the clinically defined variants of SALS and PLS.

**Methods:**

Microarray analysis was performed on fibroblast RNA and differentially expressed genes identified. Genes in enriched biological pathways were validated by quantitative PCR and functional assays performed to establish the effect of altered RNA levels on the cellular processes.

**Results:**

Gene expression profiling demonstrated that whilst there were many differentially expressed genes in common between SALS and PLS fibroblasts, there were many more expressed specifically in the SALS fibroblasts, including those involved in RNA processing and the stress response. Functional analysis of the fibroblasts confirmed a significant decrease in miRNA production and a reduced response to hypoxia in SALS fibroblasts. Furthermore, metabolic gene changes seen in SALS, many of which were also evident in PLS fibroblasts, resulted in dysfunctional cellular respiration.

**Conclusions:**

The data demonstrate that fibroblasts can act as cellular models for ALS and PLS, by establishing the transcriptional changes in known pathogenic pathways that confer subsequent functional effects and potentially highlight targets for therapeutic intervention.

## Introduction

Amyotrophic lateral sclerosis (ALS) is a fatal adult onset neurodegenerative disease characterized by the relentlessly progressing degeneration of both upper and lower motor neurones (UMN and LMN) of the motor cortex, brainstem and spinal cord. Symptom onset is predominantly in the upper or lower limbs, or in the bulbar musculature; in rare instances, it presents in the respiratory muscles [1]. The disease survival is usually 2–3 years from symptom onset, though 5% of cases may survive over 10 years [2]. ALS is the most common variant of a spectrum of motor neurone diseases (MND), encompassing syndromic variants including primary lateral sclerosis (PLS), progressive muscular atrophy (PMA) and progressive bulbar palsy (PBP). The variants differ primarily in which type of motor neurone degenerates and the rate of disease progression.

PLS, which accounts for 2.9–3.7% of MND, is clinically defined following 4 years of exclusive UMN involvement [3]. In contrast to ALS, PLS is associated with a more benign disease course of up to 17 years [4]. Although pathological hallmarks of ALS such as TDP-43 immunoreactive ubiquitinated inclusions have been observed in PLS [5], due to the rarity of the cases and less aggressive disease progression, there has been a paucity of studies investigating aetiological mechanisms in PLS specifically. There is, however, substantial overlap in the clinical presentation between MND variants, as cases presenting with PLS can go on to develop LMN degeneration accompanied by a rapidly progressing phenotype, with time to LMN involvement indicative of disease duration [4,6].

In familial ALS (FALS), which accounts for 5% of all ALS cases, mutations in genes such as *SOD1*, *TARDBP*, *FUS*, *VAPB*, *VCP* and *ALS2*, have provided invaluable insights into pathogenic mechanisms of disease. Whilst the function of the recently identified C9ORF72 protein and the effect of the expanded G_4_C_2_ repeat in intron 1 have yet to be established [7,8], the repeat in *C9ORF72* has recently been estimated to account for 39% of FALS and 4–8% of SALS cases [9]. The clinical and genetic heterogeneity of ALS, presents a significant hurdle in modelling the sporadic disease, and despite having an estimated heritability of 0.61 [10], a robust marker for SALS has not yet been identified. In contrast to ALS, PLS is generally sporadic.

The clinical and pathological picture of FALS and SALS are largely similar with motor neurone death occurring as a culmination of complex gene-environment interactions and the activation of multiple pathophysiological mechanisms including oxidative stress, dysregulation of RNA processing, mitochondrial dysfunction, protein aggregation, excitotoxicity, disordered axonal transport and inflammation [11]. However, preclinical *in vitro* and *in vivo* trials focusing on FALS models of the disease, which may not adequately model sporadic disease, have led to a failure in translation of therapies into clinical benefits in ALS [12]. Thus, there is a need for surrogate models of disease that are representative of the genetic and environmental background of individual patients. The aim of this study was to determine the expression profiles of a surrogate tissue from SALS and PLS patients and to establish if these fibroblasts can act as a cellular model for these disease states. The use of fibroblasts from non-genetic variants allows us to explore the transcriptional changes underlying dysregulated processes that recapitulate central nervous system (CNS) pathogenic mechanisms and additionally identify biological processes that underlie the pathological distinction between clinically defined variants of ALS and PLS.

Gene expression profiling in cell culture models and post-mortem tissue of transgenic animals and ALS patients has provided valuable insights into ALS pathogenesis [13]. The ease of obtaining and cell culturing skin fibroblasts make them a valuable model system to study neurodegenerative mechanisms. Previous reports have demonstrated abnormalities in mechanisms relevant to neurodegeneration such as response to oxidative stress, alterations in energy metabolism and calcium handling in fibroblasts cultured from Alzheimer's disease, Parkinson's disease and ALS patients 14–17. By using gene expression profiling in fibroblasts, we have identified many transcriptional alterations in common between SALS and PLS fibroblasts, whilst also determining that SALS fibroblasts showed significantly more changes in genes involved in RNA processing and the response to stress. These data demonstrate a proof-of-principle for the usefulness of fibroblasts as a disease model to study pathophysiological mechanisms, to identify targets amenable to therapeutic intervention and to screen potential disease modifying drugs in SALS and PLS.

## Materials and methods

### Patient details

Experiments were carried out using fibroblasts obtained from SALS patients, PLS patients and healthy, neurologically normal controls (Table S1). Patients with SALS met the El Escorial criteria for definite or probable ALS. Patients were classified as PLS following 4 years of exclusive UMN involvement. None of the patients included in this study carried mutations in the most common ALS genes, *SOD1*, *TARDBP*, *FUS* and *C9ORF72*. The average age at disease onset in the ALS cohort was 57 years (range 38–74 years) with average disease duration of 3 years (range 1–6 years) at the time of sampling. In the PLS cohort, the average age at disease onset was 55 years (range 45–70 years), with an average disease duration of 13 (range 9–20 years) at the time of sampling. The average age of controls was 53 years (range 37–76 years).

### Fibroblast cultures

Skin biopsies were obtained from the forearm of subjects under sterile conditions after informed consent in accordance with guidelines set by the local ethics committee. Fibroblast cell cultures were established at the Metabolic Biochemistry Unit, Sheffield Children's Hospital NHS Foundation Trust. Monolayers of primary fibroblast cell cultures were routinely maintained in T75 flasks with fibroblast cell culture medium Ham's F-10 Media (Gibco, Life Technologies, Paisley, UK) containing 10% foetal calf serum (Biosera, Labtech International, Uckfield, UK), 1% penicillin-streptomycin antibiotic solution, 0.25% holo-transferrin and 2.5 ng epidermal growth factor (all from Sigma, Gillingham, UK), in incubators at 37°C supplemented with 20% O_2_ and 5% CO_2_ unless stated otherwise_._ Cultures at 70–80% confluency were serially passaged by washing with PBS and treatment with trypsin (0.5 μg/l)-EDTA (0.2 μg/l). Fibroblast cell lines were maintained and used up to passage 11. For the hypoxia experiments, fibroblasts from control and patients were cultured for an additional 18 h simultaneously in normoxia conditions (20% O_2_) as well in a 1% O_2_ hypoxic chamber.

### RNA extraction, amplification, fragmentation and labelling

RNA was isolated from 6 SALS, 6 PLS and 6 control fibroblast cultures using the RNeasy Mini Kit (Qiagen, Manchester, UK) as per the manufacturer's protocol. RNA was linearly amplified and biotin labelled using the One-Cycle Affymetrix Labelling Kit (Affymetrix, High Wycombe, UK) as per the manufacturer's protocol. Biotin-labelled cRNA was fragmented to 35–200 bp fragments by heating to 95°C for 35 min with a fragmentation buffer (Affymetrix) and subsequently hybridized overnight at 42°C to Human U133 Plus 2.0 GeneChips as per the Eukaryotic Target Hybridisation protocol (Affymetrix). GeneChips were stained and washed in a GeneChip Fluidics Station 400 (Affymetrix). The fluorescence intensity of hybridized transcripts was determined with the high-resolution laser of the GeneChip 3000 Scanner (MegAllele system, Affymetrix).

### Microarray analysis

Background correction, normalization and summarization of GeneChip fluorescence intensities to derive signal intensities by incorporating a scaling and weighting of experimentally observed inherent thermodynamic properties of every contributing PM/MM probe pair was carried out using PLIER (Probe Logarithmic Intensity Error) algorithm in GeneSpring GX 11 (Agilent Technologies, Wokingham, UK). One-way anova and Tukey post hoc test was applied to detect differential expression across the SALS, PLS and healthy control groups. Transcripts were defined as differentially expressed if they differed between groups with a fold change of >1.5 fold and a *P*-value of <0.05. Where a gene was represented by more than one transcript, the most significant transcript was retained for further analysis. Differentially expressed transcripts were classified according to their biological function based on GO terms identified by NetAffx™ (Affymetrix) and DAVID (Database for Annotation, Visualisation and Integrated Discovery) [18]; DAVID was then used to identify Gene Ontology (GO) biological processes and molecular functions enriched in the list of differentially expressed transcripts.

### Q-PCR validation

12.5 ng of total RNA from 11 ALS cases, 6 PLS cases and 10 controls (Table S1) was reverse transcribed using Superscript III and oligo-dT primers as per the manufacturers protocol (Invitrogen, Life Technologies, Paisley, UK) and used along with 1× Brilliant II SYBR Green PCR Master Mix (Stratagene, Agilent Technologies, Cheadle, UK) and optimized concentrations of forward and reverse primers in each assay (Table S2). The assays were run on an MX3000P Real-Time PCR system (Stratagene) with appropriate RT negative reactions without Superscript III. For each gene of interest, the fluorescent signal intensity was analysed using the MxPro software (Stratagene) and its gene expression value normalized to housekeeping gene beta-actin (*ACTB*) using the 2^ΔΔCt^ method (ABI PRISM 7700 Sequence Detection System protocol, Applied Biosystems, Warrington, UK). The *ACTB* gene was chosen as a suitable housekeeping gene due to its stable expression across all patient and control fibroblasts on the microarray chips. Expression levels for each gene are expressed relative to their expression levels in control fibroblasts. Statistical significance of expression levels between disease and controls was assessed on applying an unpaired Student's *t*-test using GraphPad Prism 6 (GraphPad Software Inc., California, USA).

### Metabolic measurements using Seahorse assays

Fibroblasts from 6 SALS patients and 9 controls (Table S1) were plated at 50 000 cells/well in a 24 well Seahorse cell culture plate (Seahorse Bioscience, Copenhagen, Denmark) in 250 μl minimal media (PAA) supplemented with 10% FCS gold (PAA), 2 mM glutamine (Lonza, Basel, Switzerland), 50 μg/ml uridine (Sigma), 100× vitamins (Lonza 5 ml in 500 ml), 100× amino acids (Lonza 5 ml in 500 ml), sodium pyruvate (Lonza 5 ml in 500 ml) and penicillin/streptomycin (Lonza 5 ml in 500 ml 5000 U/ml). The cells were incubated at 37°C/5% CO_2_ overnight. The following day the media was removed and the cells were washed with 1000 μl XF Assay Media pH 7.4 (Seahorse Bioscience) supplemented with 1 mM sodium pyruvate, 2 mM glutamine and 1mg/ml D-glucose (Sigma). Cells were incubated at 37°C in a non CO_2_ incubator for 1 h in a total volume of 675 μl of XF Assay media. Meanwhile, a 24 well microplate was loaded with 2.5 mg/ml oligomycin (Sigma), final concentration 0.25 mg/ml. 5.0 μM FCCP (Sigma), 0.5 μM final concentration and 5.0 μM rotenone (Sigma), final concentration 0.5 μM. All were made up in assay media as described above. The microplate was calibrated in a Seahorse XF24 analyser prior to addition of the cell culture plate. Three basal oxygen consumption rate (OCR) and extracellular acidification rate (ECAR) measurements were recorded prior to addition of the mitochondrial inhibitors. The cells were measured three times for 3 min each. Cell number was normalized by addition of 2.5 μM calcein (Invitrogen) incubated with the cells for 60 min and fluorescence measured on a Fluorostar Omega plate reader (BMG Labtech, Ortenberg, Germany) at Ex485nm/Em520nm.

### MicroRNA profiling of fibroblasts

Total RNA was extracted from 6 SALS and 6 control T75 fibroblast culture flasks using the mirVana isolation kit (Applied Biosystems) according to the manufacture's protocol. TaqMan® Low Density Arrays (TLDAs) were used to measure the expression level of 377 microRNAs (miRNAs) (Applied Biosystems). Briefly, extracted RNA was used in megaplex reverse transcription reactions containing miRNA specific stem loop primers (Applied Biosystems). Following subsequent pre-amplification, the expression of miRNA was measured using Q-PCR with TLDA cards (Human A v3.0). The card data were analysed on an ABI 7900HT Q-PCR system using Sequence Detection System (SDS) software v2.3 according to the manufacturer's recommended conditions. Manual inspection of amplification plots and preliminary data analysis were performed using SDS RQ manager v1.2 and Data Assist software v2.0 respectively (Applied Biosystems). Relative expression of mature miRNAs was calculated using the competitive CT 2^ΔΔCt^ method, with stably expressed miRNAs across all samples, (as identified using NormFinder algorithm [19], acting as controls to normalize the data.

### Immunoblotting for hypoxic response in fibroblasts

Total protein was extracted by washing cells twice in PBS and scraping into 100 μl cell lysis RIPA buffer (150 mM NaCl, 1% IGEPAL® CA-630, 0.5% sodium deoxycholate, 0.1% SDS, 50 mM Tris pH 8.0, plus protease inhibitor cocktail) (Sigma) at 4°C. Protein concentration was determined by the colorimetric Bradford assay using Coomassie Brilliant Blue dye (Bio-Rad, Hemel Hempstead, UK). 80 μg of total protein along with pre-stained protein ladder (Bioline, London, UK) was separated on 10% sodium dodecyl sulphate polyacrylamide gel (SDS-PAGE). Protein samples were transferred overnight onto polyvinylidene difluoride (PVDF) membrane (Millipore, Watford, UK) and blocked in PBS-T and 5% (w/v) dried skimmed milk. The blots were then probed in PBST plus milk with mouse polyclonal anti HIF-1α (R&D Biosciences, Abingdon, UK) diluted 1:500, rabbit polyclonal anti-PHD2 (Abcam, Cambridge, UK) diluted 1:1000 and rabbit polyclonal anti-tubulin (Abcam) diluted 1:5000. To detect HIF-1α, the blot was incubated for 4 h at room temperature; to detect PHD-2 and tubulin incubation was for 1 h at room temperature. Subsequently, each blot was probed for 1 h at room temperature with peroxidase conjugated goat anti-mouse secondary antibody diluted 1:6000 (for HIF-1α) or goat anti-rabbit secondary antibody diluted 1:4000 (for PHD-2 and tubulin). Antibody bound to protein was detected by enhanced chemiluminescence (ECL) kit (Amersham, GE Healthcare Life Sciences, Little Chalfont, UK) as per the manufacturer's protocol. Protein quantification was carried out using GBox-HR Gel Doc system (Syngene, Cambridge, UK).

### VEGF ELISA

To quantify VEGF protein levels, supernatants were collected, spun down to remove cellular debris and tested for VEGF protein using Quantikine VEGF ELISA kit (R&D Biosciences) as per the manufacturer's protocol.

## Results

### Microarray analysis

**Figure 1:**
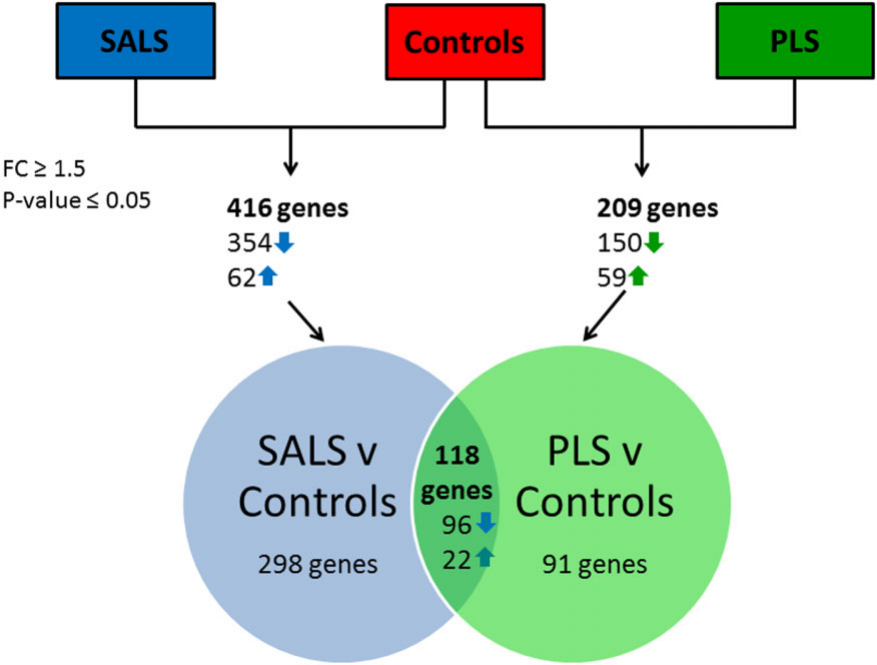
Gene expression analysis showing the number of differentially expressed genes pairwise comparisons between the SALS *vs.* control fibroblasts and PLS *vs.* controls fibroblasts. The number of genes that are differentially expressed in common between ALS and PLS *vs.* controls are also shown. (Differentially expressed genes are defined as those showing an FC of ≥1.5 and a *P*-value of ≤0.05 between the disease group and control fibroblasts.)

The transcriptional profiles generated from all fibroblasts showed comparable quality control parameters with an average of 42.3% of transcripts called as present in SALS fibroblasts, 42.4% in PLS fibroblasts and 42.3% in control fibroblasts (CEL files have been submitted to the Gene Expression Omnibus Repository, Accession GSE56808). Comparative analysis using GeneSpring identified 416 significantly differentially expressed transcripts in SALS (Table S3). A more subtle 209 transcripts in PLS fibroblasts were differentially expressed compared to controls (Table S4). The majority of transcripts in the SALS and PLS fibroblasts showed a decrease in expression, compared to controls and of those genes differentially expressed in PLS, 118 transcripts (56%) were in common and were regulated in the same direction in SALS fibroblasts (Figure 1).

Differentially expressed transcripts were functionally categorized using GO terms identified by NetAffx™ and DAVID. Of those transcripts differentially expressed in SALS fibroblasts, there were a large number of genes involved in transcription, signalling, metabolism and RNA processing (Table 1) and this was supported by analysis in DAVID, which demonstrated a highly significant enrichment of biological processes involving ‘transcription’, several ‘regulation of transcription’ related GO terms including ‘regulation of gene expression, epigenetic’ and ‘glucose homeostasis’ (Table 2). In PLS fibroblasts, transcription, signalling and metabolism again showed the largest number of genes differentially expressed, similar to the SALS fibroblasts (Table 1). However, there was a striking decrease in the number of genes involved in RNA processing and response to stress compared to SALS, whilst more cell adhesion genes were differentially expressed in the PLS fibroblasts. DAVID analysis identified the ‘response to estrogen stimulus’, as the most significantly enriched in the PLS fibroblasts (Table 3), though 5 of the 9 genes in this category were also altered in SALS fibroblasts and of the 20 genes in the wider GO term ‘response to organic substance’ only 7 were specific for PLS. Thus, as demonstrated from the initial categorization, SALS fibroblast gene profiles show more significant and specific gene expression changes compared to PLS fibroblasts.

**Table 1:** Biological functions altered in SALS and PLS fibroblasts and number of genes annotated for each process following categorization based on terms identified by NetAffx™ and DAVID

SALS fibroblasts		PLS fibroblasts		SALS *vs.* PLS
Biological function	No. of genes	Biological function	No. of genes	Ratio of genes differentially expressed
Transcription	63	Transcription	25	2.52
Signalling	41	Signalling	26	1.58
Metabolism	30	Metabolism	25	1.20
RNA processing	28	RNA processing	8	3.50*
Protein catabolism	15	Protein catabolism	6	2.50
Cytoskeleton	14	Cytoskeleton	6	2.33
Response to stress	14	Response to stress	2	7.00*
Cell cycle	12	Cell cycle	6	2.00
Cell adhesion	10	Cell Adhesion	17	0.59
Immune response	7	Immune response	7	1.00
Protein transport	7			
Apoptosis	5	Apoptosis	3	1.67
Ion transport	5	Ion transport	4	1.25
Protein translation	3	Protein transport	4	0.75
Protein processing	2			
Miscellaneous	35	Miscellaneous	14	2.50
Unknown	125	Unknown	56	2.23

The relative number of genes differentially expressed in SALS compared with PLS fibroblasts is also provided.

* Response to stress and RNA processing are the categories which are altered most in SALS, compared with the PLS fibroblasts.

**Table 2:** Top 20 gene ontology biological processes altered in SALS fibroblasts as determined by the functional annotation chart using the DAVID analysis software

Gene ontology biological process	No. of genes	% differentially expressed genes	*P*-value
GO:0006350∼transcription	84	22.05	7.13E-10
GO:0045449∼regulation of transcription	96	25.20	1.65E-09
GO:0051252∼regulation of RNA metabolic process	73	19.16	1.21E-08
GO:0006355∼regulation of transcription, DNA-dependent	69	18.11	1.39E-07
GO:0006357∼regulation of transcription from RNA polymerase II promoter	34	8.92	1.45E-05
GO:0010608∼posttranscriptional regulation of gene expression	15	3.94	1.15E-04
GO:0040029∼regulation of gene expression, epigenetic	9	2.36	1.73E-04
GO:0042593∼glucose homeostasis	7	1.84	4.66E-04
GO:0033500∼carbohydrate homeostasis	7	1.84	4.66E-04
GO:0010605∼negative regulation of macromolecule metabolic process	30	7.87	5.21E-04
GO:0016071∼mRNA metabolic process	19	4.99	6.26E-04
GO:0010629∼negative regulation of gene expression	23	6.04	7.01E-04
GO:0010558∼negative regulation of macromolecule biosynthetic process	24	6.30	8.74E-04
GO:0043009∼chordate embryonic development	17	4.46	0.0013
GO:0009792∼embryonic development ending in birth or egg hatching	17	4.46	0.0014
GO:0042127∼regulation of cell proliferation	30	7.87	0.0015
GO:0032350∼regulation of hormone metabolic process	4	1.05	0.0016
GO:0009890∼negative regulation of biosynthetic process	24	6.30	0.0016
GO:0008380∼RNA splicing	15	3.94	0.0022
GO:0006397∼mRNA processing	16	4.20	0.0026

For each process, the number and percentage of differentially expressed genes and the statistical significance has been shown.

**Table 3:** Top 20 gene ontology biological processes altered in PLS fibroblasts as determined by the functional annotation chart using the DAVID analysis software

Gene ontology biological process	No. of genes	% differentially expressed genes	*P*-value
GO:0043627∼response to estrogen stimulus	9	4.64	2.46E-05
GO:0009719∼response to endogenous stimulus	16	8.25	5.51E-05
GO:0010559∼regulation of glycoprotein biosynthetic process	4	2.06	2.19E-04
GO:0009725∼response to hormone stimulus	14	7.22	2.72E-04
GO:0032355∼response to estradiol stimulus	6	3.09	3.51E-04
GO:0010033∼response to organic substance	20	10.31	5.01E-04
GO:0042493∼response to drug	10	5.15	7.82E-04
GO:0030217∼T cell differentiation	6	3.09	8.29E-04
GO:0033273∼response to vitamin	6	3.09	8.89E-04
GO:0051252∼regulation of RNA metabolic process	36	18.56	9.09E-04
GO:0030098∼lymphocyte differentiation	7	3.61	0.001101
GO:0006355∼regulation of transcription, DNA-dependent	35	18.04	0.0012
GO:0010560∼positive regulation of glycoprotein biosynthetic process	3	1.55	0.0012
GO:0006357∼regulation of transcription from RNA polymerase II promoter	19	9.79	0.0014
GO:0045449∼regulation of transcription	46	23.71	0.0015
GO:0048545∼response to steroid hormone stimulus	9	4.64	0.0015
GO:0042113∼B cell activation	6	3.09	0.0017
GO:0030183∼B cell differentiation	5	2.58	0.0021
GO:0014070∼response to organic cyclic substance	7	3.61	0.0025
GO:0035137∼hindlimb morphogenesis	4	2.06	0.0030

For each process, the number and percentage of differentially expressed genes and the statistical significance has been shown.

RNA processing and the stress response have been identified previously to play a role in ALS pathogenesis. We therefore hypothesized that the significantly and highly enriched differential expression of these processes in SALS fibroblasts are mimicking CNS neurodegenerative mechanisms, and involve genes that underlie the pathological distinction between SALS and PLS. In contrast, the common biological pathways of metabolism, signalling and transcription may provide a therapeutic target for both disease variants, and the greatest degree of correlation between PLS and SALS was in the metabolic genes (with 11 in common). For the purpose of this manuscript, downstream analysis of the data has focused on these three biological categories: (i) metabolism, (ii) RNA processing and (iii) response to stress, using Q-PCR validation and functional assays in SALS and control fibroblasts to evaluate the validity of fibroblasts as a cell model for SALS.

### Metabolism and energy production

**Figure 2:**
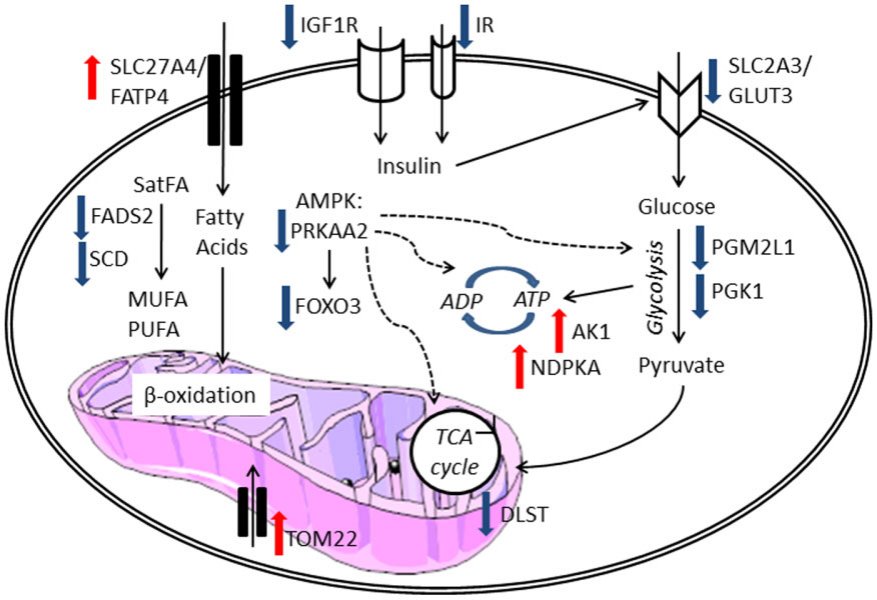
Schematic representation of the differentially expressed genes in the SALS fibroblasts compared to controls which are involved in metabolic pathways and their corresponding protein localizations within the cell. Red arrows indicate there is an increase in the expression of the gene; blue arrows indicate a decrease in gene expression.

Fibroblasts mainly rely on ATP generated via glycolysis to meet their energy requirements [14]. SALS fibroblasts exhibit significant transcriptional changes in energy metabolism genes, including those in the insulin-mediated signalling cascade, glycolytic and lipid homeostatic pathways. The expression profiling data suggest that the dysregulation of insulin signalling and glycolysis results in a compensatory increase in fatty acid oxidation to generate ATP via β-oxidation (Figure 2 and Table 4).

**Table 4:** Genes involved in metabolism, RNA processing, response to stress and cell survival which are differentially expressed in SALS fibroblasts compared to controls

Gene symbol	Gene name	Fold change	*P*-value
Metabolism			
*AGXT2L2*	Alanine-glyoxylate aminotransferase 2-like 2	−1.51	0.027
*AK1*	Adenylate kinase 1	+1.64	0.0001
*AK4/AK3L1*	Adenylate kinase 4/ adenylate kinase 3 like 1	−1.58	0.015
*B4GALT1*	UDP-Gal:beta-GlcNAc beta-1,4-galactosyltransferase 1	−2.41	0.001
*DLST*	E2 componenet of oxo-glutarate complex	−1.51	0.004
*EBP*	Emopamil binding protein (sterol isomerase)	+1.74	0.005
*FADS2*	Fatty acid desaturase 2	−3.23	0.0002
*FOXO3*	Forkhead box 3	−1.68	0.001
*IGF1R*	Insulin like growth factor-1 receptor	−2.56	0.040
*IGF2BP1*	Insulin-like growth factor 2 mRNA binding protein 1	+3.83	0.003
*IGF2BP2*	Insulin-like growth factor 2 mRNA-binding protein 2	−1.63	0.031
*IR*	Insulin receptor	−3.08	0.011
*NDUFS7*	NADH-coenzyme Q reductase	+1.54	0.008
*NDUFS8*	NADH dehydrogenase ubiquinone Fe-S 8	+1.69	0.002
*NME1/NDPKA*	Non-metastatic cells 1/ nucleoside disphosphate kinase A	+1.61	0.002
*NP*	Purine nucleoside phosphorylase	+1.56	0.042
*PDE7B*	Phosphodiesterase 7B	−1.60	0.028
*PGK1*	Phosphoglycerate kinase 1	−1.74	0.034
*PGM2L1*	Phosphoglucomutase 2-like 1	−1.62	0.010
*PPA2*	Pyrophosphatase (inorganic) 2	−1.52	0.037
*PRKAA2*	AMP-activated protein kinase alpha 2 catalytic subunit	−1.55	0.013
*PRPS2*	Phosphoribosyl pyrophosphate synthetase 2	−3.10	0.046
*SC4MOL*	Sterol-C4-methyl-oxidase like	−1.56	0.040
*SCD*	Stearoyl-CoA desaturase (delta-9-desaturase)	−1.78	0.026
*SCD5*	Stearoyl-CoA 9-desaturase	−1.97	0.007
*SLC25A36*	Solute carrier family 25, member 36	−1.72	0.003
*SLC25A37*	Solute carrier family 25, member 37	−2.49	0.026
*SLC27A4/FATP4 TP4FATP4/FATP4/*	Solute carrier family 27 (fatty acid transporter) member 4	+1.57	0.006
*SLC2A3/GLUT3*	Solute carrier family 2 (facilitated glucose transporter) member 3	−1.63	0.002
RNA processing			
*AGO1/EIF2C1*	Argonaute 1/Eukaryotic translation initiation factor 2C 1	−1.54	0.040
*AGO2/EIF2C2*	Argonaute 2/Eukaryotic translation initiation factor 2C 2	−2.22	0.011
*DGCR8*	DiGeorge syndrome critical region 8	−1.52	0.021
*DICER1*	Dicer1, Dcr-1 homolog	−2.03	0.047
*EXOSC4*	Exosome component 4	+1.53	0.004
*FUS*	Fused in sarcoma	−1.79	0.018
*G3BP1*	GTPase activating protein (SH3 domain) binding protein 1	+1.53	0.024
*HNRNPD*	Heterogenous nuclear ribonucleoprotein D	−1.64	0.006
*HNRNPM*	Heterogenous nuclear ribonucleoprotein M	−1.65	0.024
*HNRNPU*	Heterogenous nuclear ribonucleoprotein U	−1.85	0.031
*KHSRP*	FUSE-binding protein 2	−1.94	0.019
*LSM4*	U6 snRNP-associated Sm-like protein 4	+1.52	0.032
*MBNL1*	Muscleblind-like 1	−1.97	0.012
*NXF3*	Nuclear RNA export factor 3	−1.58	0.021
*PCBP2/HNRNPE2*	Poly(rC) binding protein 2/ heterogeneous nuclear ribonucleoprotein E2	−2.14	0.039
*PRMT7*	Protein arginine methyltransferase 7	+1.54	0.011
*QK1*	Quaking, mouse homolog of	−1.53	0.011
*RBMS3*	RNA binding motif protein, single strand-interacting 3	−2.03	0.019
*RBPMS*	RNA binding protein gene with multiple splicing	−1.57	0.004
*SFRS18*	Splicing factor, arginine/serine-rich 18	−1.62	0.006
*SNRPA1*	Small nuclear ribonucleoprotein polypeptide A’	−1.72	0.020
*TAF15*	TAF15 RNA polymerase II, TAT box binding protein	−1.65	0.034
*TAF9B*	TAF9B RNA polymerase II, TATA box binding protein (TBP)-associated factor	−2.33	0.006
*TRA2A*	Transformer 2 alpha homolog (Drosophila)	−2.57	0.017
*WHSC2*	Wolf-Hirschhorn syndrome candidate 2	−1.54	0.013
*WTAP*	Wilms tumour 1 associated protein	−2.19	0.048
*ZFP36L2*	Zinc finger protein 36, C3H type-like 2	−1.65	0.049
Response to stress (and cell survival)			
*AKR1C4*	Aldo-keto reductase family 1, member C4	+1.76	0.024
*ARNT/HIF1B*	Aryl hydrocarbon receptor nuclear translocator	−2.4	0.017
*ARNT2*	Aryl hydrocarbon receptor nuclear translocator 2	−1.74	0.008
*BIRC5*	Baculoviral IAP repeat-containing protein 5	+1.65	0.020
*DDAH1*	Dimethylarginine dimethylaminohydrolase 1	−3.21	0.020
*DUSP1*	Dual specificity phosphatase 1	+1.82	0.028
*EP300*	Histone acetyltransferase p300	−1.87	0.026
*FAC1*	Foetal Alzheimer antigen	−2.05	0.009
*FOS*	v-fos FBJ murine osteosarcoma viral oncogene homolog	+2.43	0.004
*GPX1*	Glutathione peroxidase 1	+1.57	0.039
*HIF1A*	Hypoxia inducible factor 1A	−1.65	0.032
*MCL1*	Myeloid cell leukemia sequence 1 (BCL2-related)	−1.64	0.020
*MINK1*	Misshapen-like kinase 1	−1.58	0.003
*PDCD6*	Programmed cell death 6	−1.67	0.043
*PENK*	Enkephalin A	+4.24	0.012
*PHLDA1*	Pleckstrin homology-like domain, family A, member 1	+1.60	0.014
*RBBP6*	Retinoblastoma-binding protein 6	−1.60	0.0002
*STEAP3*	STEAP family member 3	+1.52	0.001
*ZAK*	Leucine zipper and sterile alpha motif-containing kinase	−2.02	0.038

The full list of genes differentially expressed in SALS fibroblasts is provided in Table S3.

#### Insulin signalling

**Figure 3:**
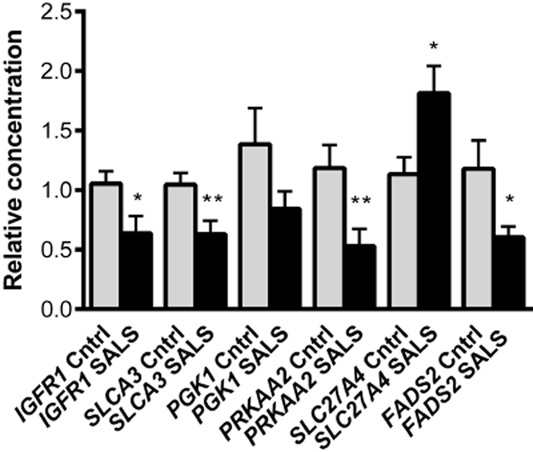
Q-PCR validation of genes involved in glucose and fatty acid metabolism in SALS fibroblasts (*n* = 11) compared to control fibroblasts (*n* = 11): *IGF1R*, *SLC2A3/GLUT3*, *PGK1*, *PRKAA2*, *SLC27A4/FATP4* *and* *FADS2*. Error bars indicate SEM. *P ≤ 0.05; **P < 0.01.

Binding of insulin or insulin-like growth factor to the insulin receptor (IR) and insulin-like growth factor receptor (IGF1R) activates a signalling pathway which leads to localization of the glucose transporters at the plasma membrane ready for glucose uptake, and increased transcription of glucose and lipid metabolism genes in the nucleus. In the fibroblasts of SALS patients the expression of *IR* and *IGF1R* were both decreased (*IR* −3.08; *IGF1R* −2.56) and the decrease in *IGF1R* was consequently confirmed by Q-PCR (*P* = 0.03) (Figure 3). In addition, there was differential expression of two proteins which bind to the insulin-like growth factor 2 (IGF2) mRNA, IGF2 mRNA binding proteins 1 and 2 (*IGF2BP1* +3.8 and *IGF2BP2* −1.6). These were originally identified to regulate IGF2 translation, with IGF2BP1 inhibiting translation, whilst IGF2BP2 enhanced translation. However, subsequent work has shown that IGF2BPs have many mRNA targets, resulting in either inhibition or enhancement of translation or inhibition of mRNA decay [20].

#### Glycolysis

In SALS fibroblasts, there was a decrease in glucose transporter 3 *(SLC2A3/GLUT3* −1.63), the glucose transporter which is highly expressed in neurones and which has the highest glucose affinity of the four transporters [21]. The differential expression of *SLC2A3/GLUT3* was confirmed by Q-PCR (*P* = 0.01) (Figure 3). There was decreased expression of glucose metabolism genes including phosphoglucomutase-2 like 1 (*PGM2L1* −1.62), which controls the rate of glycolytic flux, and phosphoglycerate kinase (*PGK1* −1.74), which catalyses the first ATP producing step of glycolysis, although Q-PCR validation of *PGK1* did not reach statistical significance (*P* = 0.12) (Figure 3). Transcriptional changes were also seen in cytosolic adenylate kinase 1 (*AK1* +1.64) and nucleoside disphosphate kinase A (*NDPKA/NME1* +1.61), which along with *PGK1* are involved in a tightly regulated enzymatic network that permits the transfer of high-energy phosphoryl groups (in the form of ATP) between energy producing and energy consuming sites within the cell [22]. The increased expression of *AK1* and *NDPKA* may be an attempt to compensate for the reduction in ATP caused by reduced *PGK1* expression. In contrast, the processed pseudogene AK4 (also known as AK3L1) was decreased (*AK4* −1.53).

The tricarboxylic acid (TCA) cycle is predicted to be disrupted by a decrease in dihydrolipoamide s-succinyl transferase (*DLST* −1.5), which encodes the E2 component of the α-ketoglutarate enzyme of the TCA cycle. In addition, the *PRKAA2* subunit of AMP activated protein kinase (*PRKAA2* −1.55), which acts as a central checkpoint monitoring systemic and cellular energy status and its downstream target forkhead box O3 (*FOXO3* −1.68) were both decreased. *PRKAA2* was subsequently validated by Q-PCR (*P* = 0.01) (Figure 3). Thus the collective down-regulation of insulin signalling along with key glycolytic enzymes, indicates a dysfunction in glycolysis driven energy generation in SALS fibroblasts.

#### Fatty acid oxidation

SALS fibroblasts showed an increased expression of solute carrier 27 family A member 4 (*SLC27A4/FATP4* +1.57), the principal fatty acyl-CoA synthetase in skin fibroblasts for the activation of very long chain fatty acids and their entry into the mitochondrial β-oxidation pathway [23]. This was confirmed by Q-PCR (*P* = 0.02) (Figure 3). Translocase of outer mitochondrial membrane 22 (*TOMM22 +*1.69 fold), which is a part of the protein transport machinery needed for the mitochondrial import of nuclear proteins involved in oxidative phosphorylation and lipid metabolism, was also increased in SALS fibroblasts. In contrast, fatty acid desaturases, which are responsible for the conversion of saturated fatty acids to mono and polyunsaturated fatty acids (MUFA and PUFA), were decreased in SALS fibroblasts *(FADS2* −3.23, *SCD* −1.78 and *SCD5* −1.78). The MUFA and PUFA are constituents of phospholipids, triglycerides and cholesterol esters that are essential constituents of cell membranes, vital stores of energy and regulators of signalling mechanisms [24]. Differential expression of *FADS2* was confirmed by Q-PCR (*P* = 0.03) (Figure 3). These transcriptional changes suggest that SALS fibroblasts may be correcting for the energy deficit in carbohydrate metabolism by shutting down fatty acid synthesis and driving ATP production through fatty acid oxidation.

### RNA processing

RNA processing is an important level of post-transcriptional regulation of gene expression and it encompasses a wide range of spatiotemporally linked functions including RNA splicing, nucleo-cytoplasmic transport, translational silencing and RNA degradation, as well as non-coding RNA production. SALS fibroblasts exhibit a specific and marked down-regulation of multiple transcripts involved in different RNA metabolic processes (Table 4).

#### RNA binding and RNA splicing proteins

**Figure 4:**
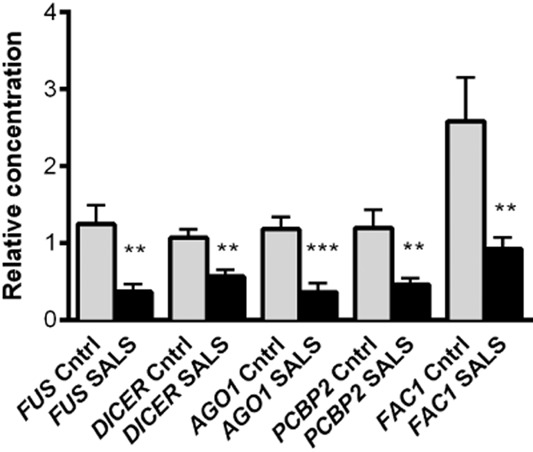
Q-PCR validation of genes involved in miRNA processing and oxidative stress in SALS fibroblasts (*n* = 11) compared to control fibroblasts (*n* = 11): *FUS*, *DICER*, *AGO1*, *PCBP2* and *FAC1*. Error bars indicate SEM. **P ≤ 0.01, ***P ≤ 0.001.

Of particular interest in this category was the decreased expression of the FALS-associated nucleo-cytoplasmic shuttling RNA binding protein fused in sarcoma (*FUS* −1.79), which is involved in RNA splicing and regulation of transcription. This differential expression was subsequently validated by Q-PCR (*P* = 0.003) (Figure 4). Decreased expression of other transcripts involved in splicing included the small nuclear ribonucleoprotein polypeptide A1 (*SNRPA1* −1.72), a component of the U2 snRNP particle, splicing factor arginine/serine-rich 18 (*SFRS18* −1.62) and the large heterogeneous nuclear ribonucleoproteins HNRNPD, HNRNPM and HNRNPU (*HNRNPD* −1.64; *HNRNPM* −1.65; *HNRNPU* −1.85). Other RNA binding proteins that showed decreased expression were RNA binding protein with multiple splicing (*RBPMS* −1.57), RNA binding motif, single stranded interacting protein 3 (*RBMS3* −1.65) and Wilms tumour associated protein 1 (*WTAP1* −2.19), which is known to associate with splicing factors in nuclear speckles [25]. Interestingly, RBPMS interacts with QK1, encoded by the human homolog of the mouse quaking gene, which is also decreased (*QK1* −1.53) and involved in RNA splicing, export and mRNA stability. In contrast to the extensive decreased expression of other RNA metabolism genes, expression of the ribosomal RNA processing protein 41/exosome component 4, which is involved in degradation of mRNA containing AU-rich elements in the 3′UTR, was increased (EXOSC4 +1.53).

#### miRNA processing

An important aspect of post-transcriptional regulation is controlled by microRNAs (miRNAs) that bind target mRNAs, thereby either tagging them for degradation or for translation inhibition [26,27]. During miRNA synthesis, the pri-miRNA is transcribed by RNA polymerase II/III and then undergoes sequential processing; firstly by the microprocessor complex comprising of nuclear ribonuclease III (RNASEN/DROSHA) and DiGeorge syndrome critical region 8 (DGCR8), resulting in the formation of a pre-miRNA, which is then exported out of the nucleus to the cytoplasm by exportin 5, where it is then processed by the endoribonuclease DICER complex to produce a double stranded miRNA. Whilst the complementary strand is degraded, the mature miRNA is loaded onto the RNA-induced silencing complex (RISC) alongside argonaute proteins (AGO/EIF2C), in order to bind to target mRNA molecules and inhibit translation [28].

SALS fibroblasts demonstrated decreased expression of key transcripts involved in miRNA processing, including the RNA polymerase II enzyme *TAF15* (−1.65), the microprocessor complex gene *DGCR8* (−1.52), the gene encoding *DICER1* (−1.62) and the RISC proteins *AGO1/EIF2C1* (−1.54) and *AGO2/EIF2C2* (−2.22). The differential expression of *DICER* (*P* = 0.001) and *AGO1* (*P* = 0.0004) were confirmed by Q-PCR (Figure 4). Additionally, the KH-type splicing regulatory protein (KHSRP/Fuse binding protein 2), which is a component of both DICER and DROSHA complex that optimizes the positioning and/or recruitment of both complexes during miRNA biogenesis showed decreased expression (*KHSRP* −1.94 fold) [29] and as did the poly(Rc) binding protein 2, which interacts with DICER to promote miRNA processing [30] (*PCBP2/HNRNPE2* −2.14). This protein, which localizes to P-bodies and stress granules [31] was validated by Q-PCR (*P* = 0.007) (Figure 4).

SALS fibroblasts also exhibited alterations of other RNA binding proteins, which in contrast to the other genes in this functional category, were increased. These included GTPase activating protein (SH3 domain) binding protein 1 (*G3BP1* +1.53), which is involved in stress granule assembly, and U6 snRNA-associated Sm-like protein 4 (*LSM4* +1.52), which is associated with snRNP complexes that include the U6 snRNA.

### Response to cellular stress

SALS fibroblasts exhibit alteration of transcripts linked to several different cellular adaptive responses to physiological stresses including hypoxia and oxidative stress (Table 4). These dysregulated responses appear to be compensated by other cellular pathways which are differentially expressed in favour of a pro-survival transcriptional programme.

#### Hypoxic stress

The brain is the main consumer of energy and is therefore particularly susceptible to neuronal damage and cell death arising from oxygen deprivation [32]. The adaptive response to hypoxia, which is propagated by hypoxia-inducible transcription factors (HIF), has been linked to neuroprotection and decreased cell death of neurones. Fibroblasts from SALS fibroblasts have down-regulation of both subunits encoding the heterodimeric HIF-1 protein, HIF1A (*HIF1A* −1.65) and HIF1B (*HIF1B/ARNT* −2.40 and *ARNT2* −1.74). There is also a decrease in histone acetyltransferase p300 (*EP300* −1.87), a chromatin modifier that acts as a co-activator of HIF1A mediated transcriptional changes. The hypothesized impairment in the response to hypoxia may also be associated with the observed down-regulation of *GLUT3* and glycolytic enzymes (as described above), which are downstream targets of HIF-1 [33].

#### Oxidative stress

SALS fibroblasts exhibit alterations in genes involved in the NRF2 mediated anti-oxidant response to oxidative stress. NRF2 is a transcription factor which, under basal conditions is confined to the cytoplasm bound to KEAP1. Upon oxidative stress, NRF2 is translocated to the nucleus where it binds anti-oxidant response elements in ‘programmed cell life‘ gene promoters and initiates expression. Foetal Alzheimer antigen, a zinc finger DNA binding partner of KEAP1 which competes with NRF2 for KEAP1 binding showed decreased expression (*FAC1 −2.03*), whilst v-FOS FBJ osteosarcoma oncogene homolog (*FOS* +2.43), which forms the AP-1 transcription factor with C-JUN to inhibit NRF2 responses [34] was increased. Overall, this predicts a reduced anti-oxidant response in a disease known to exhibit increased oxidative stress. *FAC1* differential expression was confirmed by Q-PCR (*P* < 0.01) (Figure 4). In fact, the only evidence of a positive response to increased oxidative stress was an increase of glutathione peroxidase 1 (*GPX1* +1.57), which is an anti-oxidant enzyme that catalyses the degradation of hydrogen peroxide.

#### Cell survival

To counteract the increased hypoxic and oxidative stress, several compensatory responses are evident. The transcriptional increase of dual specificity phosphatase 1 (*DUSP1* +1.82), an inhibitor of the p38 pathway which is activated in primary embryonic fibroblasts following oxidative stress is increased, whilst there is a concomitant decrease of p38 activators leucine zipper and sterile alpha motif containing kinase (*ZAK* −2.02) and misshapen-like kinase 1 (*MINK1* −1.58), thereby potentially limiting p38 propagation of inflammation and apoptosis. Nitric oxide is an activator of the p38 pathway, and there is evidence to suggest NO production is reduced, with decreased expression of dimethylarginine dimethylaminohydrolase 1 (*DDAH1* −3.21), an enzyme responsible for activating neuronal nitric oxide synthase (NOS) via the degradation of asymmetric dimethylarginine (ADMA).

In addition, there were several apoptotic genes differentially expressed. The pro-apoptotic gene programmed cell death 6 (*PDCD6* −1.67) was decreased, whilst there were increases in the pro-survival genes baculoviral IAP repeat containing 5 (*BIRC5* +1.72) and pleckstrin homology-like domain family A member 1 (*PHLDA1* +1.60), mounting a survival response. In contrast, the anti-apoptotic myeloid leukaemia sequence 1 (*MCL1*), which binds to BAK to inhibit its interaction with p53 and subsequent cytochrome C release, is decreased (−1.64), whilst STEAP family member 3 (*STEAP3*), a pro-apoptotic protein downstream of p53 was increased (+1.52).

### Functional validation

#### Metabolic assessment of SALS fibroblasts

**Figure 5:**
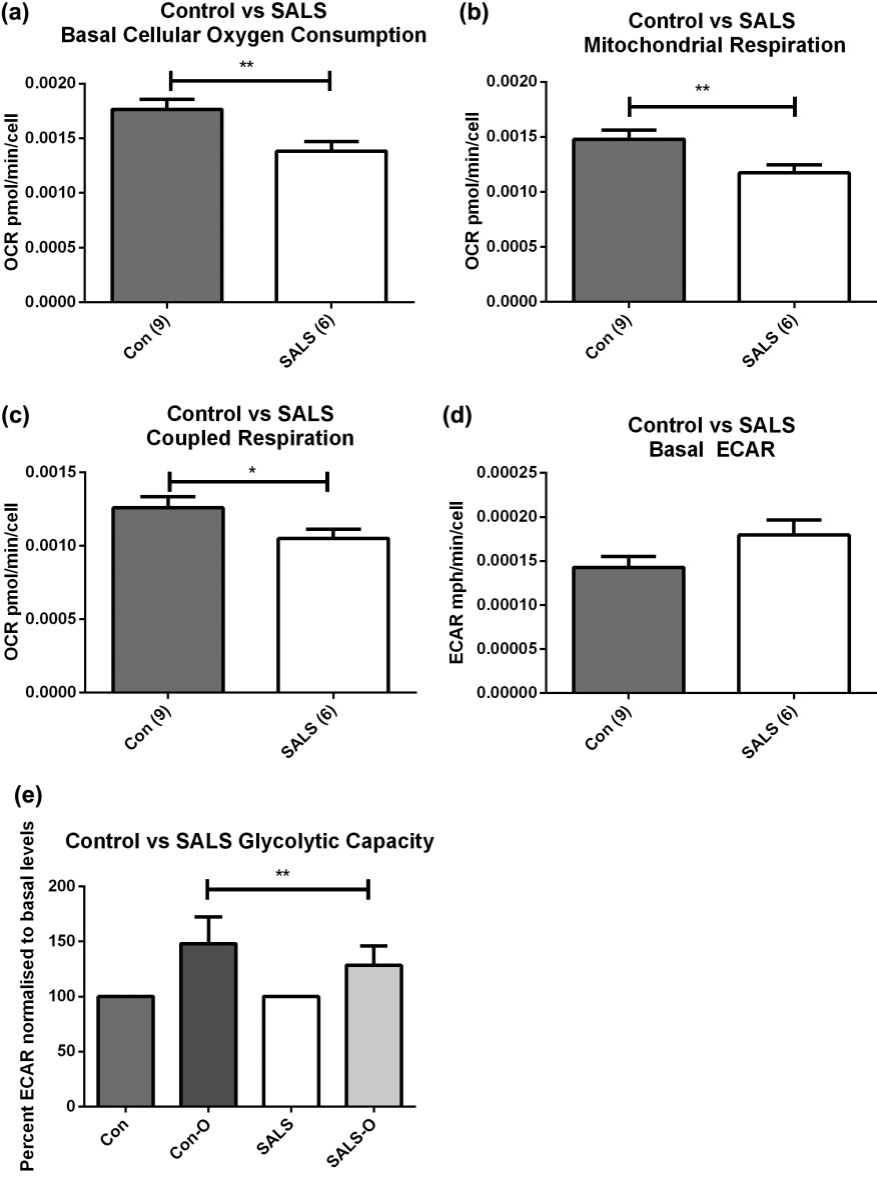
Oxygen consumption defects in SALS fibroblasts. (a) Basal Cellular Oxygen Consumption analysed in assay media supplemented with 1 mg/ml glucose, 1 mM sodium pyruvate and 2 mM glutamine. (b) Basal Mitochondrial Oxygen Consumption calculated by subtracting OCR in the presence of rotenone from total cellular OCR. **P ≤ 0.01 Mann–Whitney unpaired *t*-test. (c) Coupled respiration in SALS patients. Oxygen consumption linked to ATP synthesis was determined by injection of 0.25 μg/ml oligomycin and measuring respiration three times over a 25 min period in XF assay media. *P < 0.05 (d) Basal ECAR. (e) Glycolytic capacity, calculated by analysing the increase in ECAR in the presence of oligomycin and normalizing the increase to basal ECAR as a percentage. **P ≤ 0.01 logit transformation followed by Mann–Whitney unpaired *t*-test. *n* = 3 per patient, Error bars indicate SEM.

The differential expression of metabolic genes suggests a decrease in glycolysis in SALS fibroblasts, which is compensated by an increase in fatty acid oxidation. To determine whether these gene changes had an effect on the metabolism of live cells, the metabolic profile of SALS fibroblasts was measured under basal conditions using Seahorse XF-24 bioanalyser. This showed that SALS fibroblasts had a significantly reduced basal cellular oxygen consumption rate (COCR) compared to age matched controls (Figure 5**a**). To determine whether this drop in COCR was mitochondrial specific, the complex I inhibitor rotenone was added to the cells and the remaining OCR (which is non-mitochondrial respiration) was subtracted from total COCR. The data indicate that mitochondrial OCR (MOCR) was significantly reduced in the SALS patients (Figure 5**b**). When oligomycin was added to the cells to determine the level of mitochondrial oxygen consumption linked to ATP synthesis, the SALS patients showed a significant reduction in coupled respiration compared to controls (Figure 5**c**) though no difference was observed between controls and SALS patients in terms of uncoupled MOCR (data not shown). Basal extracellular acidification rate (ECAR) is a measure of glycolytic flux via lactate production. No significant differences were observed between controls and SALS cases (Figure 5**d**), although there was evidence of age dependent effects (data not shown). When the ATP synthase inhibitor oligomycin is added to the cells, the cells normally respond by up-regulating glycolysis to cope with the ATP energy deficit. This increase in glycolysis above basal levels gives an indication of the glycolytic capacity of the cells, a measure of glycolytic function. In the fibroblasts, when normalizing to basal levels (in order to overcome age dependent effects), the control cases were able to up-regulate glycolysis by 148%, in the presence of oligomycin, whereas the SALS cases showed significantly reduced glycolytic capacity (128%) (Figure 5**e**).

#### miRNA dysregulation in SALS fibroblasts

**Figure 6:**
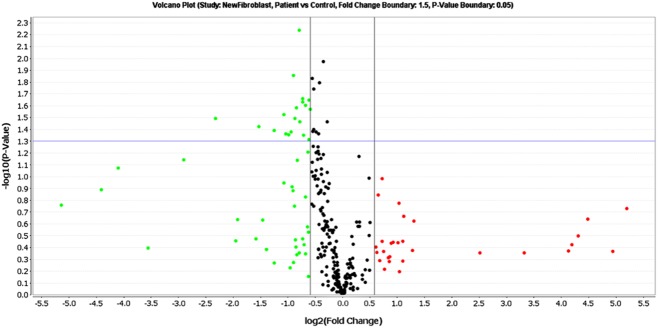
Volcano plot to display fold change *vs. P*-value from *T*-test comparison of SALS and control fibroblast miRNA expression profiles. Green dots and red dots represent miRNAs down or up regulated >1.5 fold respectively in SALS samples. The purple line represents the *P* = 0.05 level of significance.

There were significant decreases in the expression of multiple RNA processing genes, including those involved in miRNA biogenesis. To determine the effect of these changes on miRNA levels in the fibroblasts, miRNA analysis was performed on 6 SALS and 6 control fibroblast samples. Of the 227 miRNAs detected in all of the samples, 19 were significantly differentially expressed over 1.5 fold (*P* < 0.05). All of these were decreased in SALS compared to control fibroblasts (Table S5). Visualization of the miRNA expression data on a volcano plot showed there was a marked decrease in the levels of miRNAs in SALS compared to controls (Figure 6), further demonstrating an overall dysregulation of miRNA biogenesis in the SALS samples.

Of the 19 miRNAs differentially expressed, 5 have been previously described as being involved in hypoxia, through regulating or being regulated by the HIF proteins. In addition, 10 have also been found to be differentially expressed following knockdown of FUS in the SK-N-BE neuroblastoma cell line [35], whilst in contrast, miR-142-5p and miR-155 have previously been shown to be increased in ALS rodent models and human spinal cord [36,37].

#### Effect of hypoxia on SALS fibroblasts

**Figure 7:**
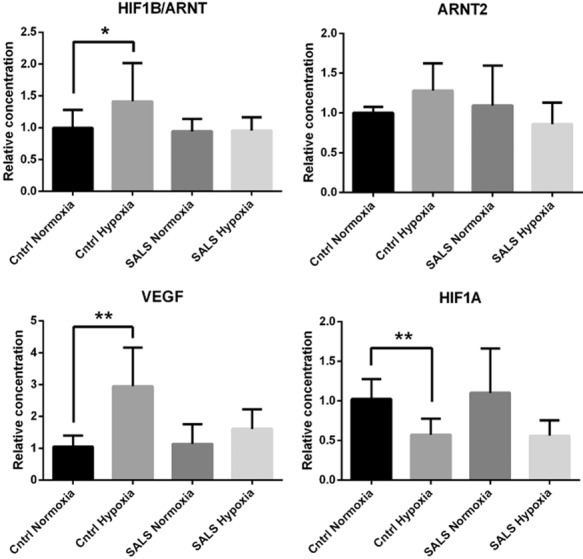
Response of fibroblasts to hypoxia. Intrinsic ability of SALS (*n* = 6) and control fibroblasts (*n* = 6) to up-regulate transcript levels of *HIF1B/ARNT*, *ARNT2*, *VEGF* and *HIF1A* following hypoxia. Error bars indicate SEM. *P ≤ 0.05, **P ≤ 0.01.

To determine the functional significance of the transcriptional changes in hypoxia response genes that were identified by microarray analysis, transcript levels of *HIF1A*, *HIF1B/ARNT* and *ARNT2*, *a*s well as the hypoxia induced gene *VEGF* were measured by Q-PCR following an 18 h incubation in 1% O_2_. The SALS fibroblasts failed to exhibit a significant increase in the intrinsic ability to up-regulate *HIF1B/ARNT*, *ARNT2* and *VEGF*, whereas control fibroblasts showed significant increases in *HIF1B/ARNT* and *VEGF* following hypoxia (Figure 7**a**–**c**). *ARNT2*, although increased in control fibroblasts, did not reach significance. Unexpectedly, however, *HIF1A* showed a significant decrease in gene expression in control fibroblasts, as well as decreased expression in SALS, following hypoxia (Figure 7**d**).

**Figure 8:**
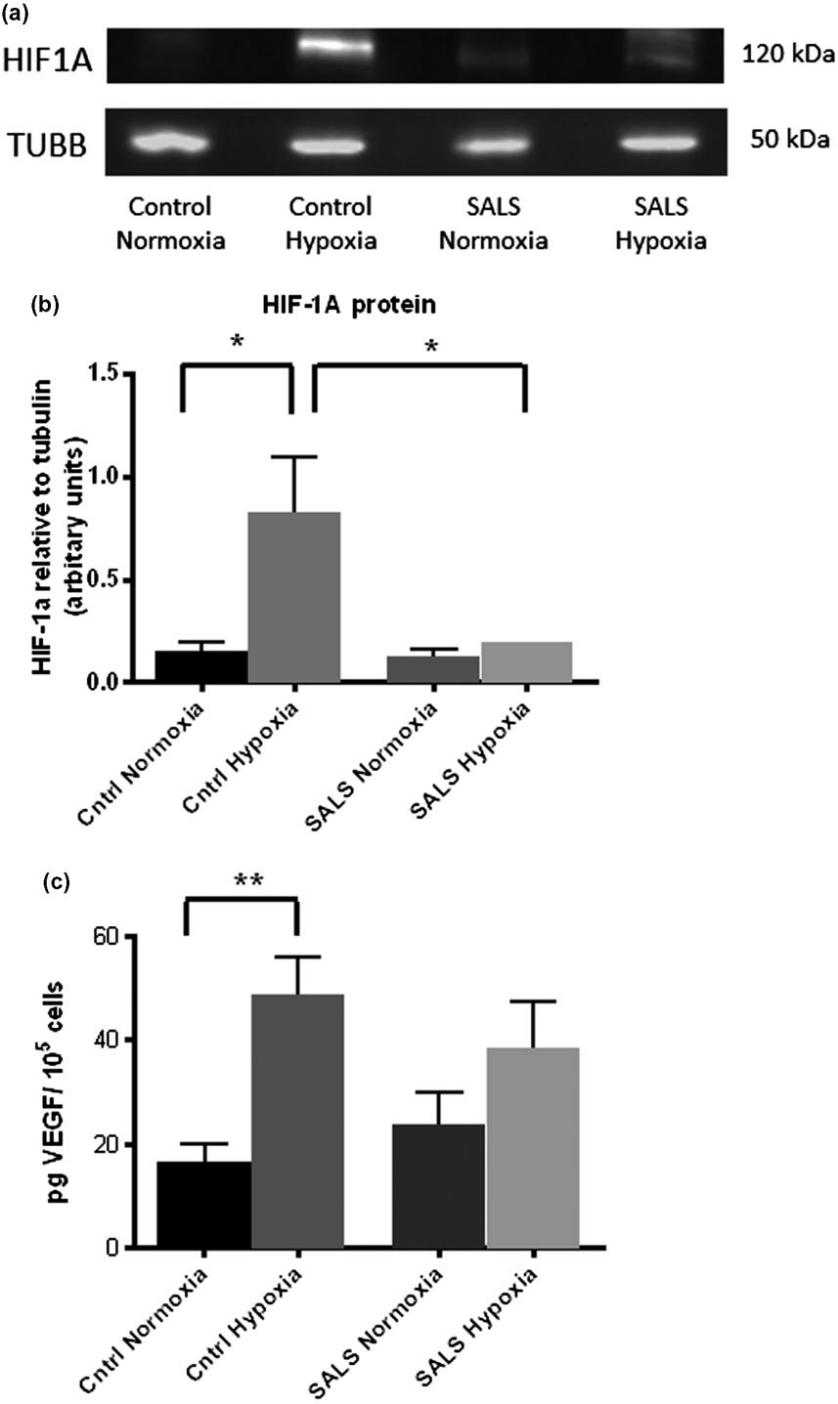
Western blotting of HIF1A protein levels and ELISA quantification of VEGF protein. (a) Representative western blot of HIF1A protein (120Kda) in control and SALS fibroblast under normal and hypoxic (1% O_2_) conditions, using beta-tubulin (50Kda) as a loading control. SALS fibroblast (lane 4) shows decreased HIF1A protein levels on comparison with control (lane 2) following hypoxia. (b) Quantification of HIF1A protein in SALS fibroblasts (*n* = 6) compared to controls (*n* = 6) under normal and hypoxic (1% O_2_) conditions. HIF1A protein levels relative to beta-tubulin were measured by densitometry and this showed significantly decreased levels of HIF1A in SALS compared to control fibroblasts following hypoxia. (c) Quantification of VEGF protein in SALS fibroblasts (*n* = 6) and controls (*n* = 6) in response to hypoxia. After hypoxia, VEGF levels were significantly higher in control (*P* = 0.02) but not patient (*P* = 0.2) fibroblasts on comparison with sample cultured in normal conditions. There was a non-significant difference in VEGF level in hypoxic controls compared to hypoxic patient fibroblasts (FC = 1.3, *P* = 0.2). Error bards indicate mean ± SEM *P < 0.05, **P < 0.01.

*HIF1A* and *HIF1B* are both usually constitutively expressed with the HIF-1 protein transcriptional activity usually regulated through post-translational modifications (PTMs) to amino acid residues on HIF1A [38]. However, we have demonstrated in SALS fibroblasts a significant decrease in gene expression of *HIF1A* and *HIF1B*, as well as the HIF-1 co-activator, p300. Therefore, assays were developed to measure the protein levels of HIF1A in hypoxic control and SALS fibroblasts. Under normal conditions, the HIF1A protein is tagged for degradation, thereby limiting accumulation in the cell. However, during hypoxia, the HIF1A protein is stabilized by PTMs, translocated into the nucleus where it dimerises with the HIF1B/ARNT transcription factor. Protein levels of HIF1A were compared between control and SALS fibroblasts by western blotting following 18 h in 1% O_2_. As expected, hypoxic control fibroblasts showed a significant increase of HIF1A protein in comparison with control fibroblasts cultured in normal conditions (FC = 6.9, *P* = 0.02), whilst hypoxic SALS fibroblasts displayed a minimal increase in HIF1A compared to SALS fibroblasts cultured in normal conditions (FC = 1.74, *P* = 0.45) (Figure 8**a**,**b**). The significant difference in HIF1A protein levels following hypoxia between controls and SALS fibroblasts (FC = 4.71, *P* = 0.04) supports the QPCR data that there is an impaired response to hypoxia in SALS fibroblasts.

To investigate this impairment further, VEGF protein levels were measured by ELISA in cell supernatants of 6 control and 6 SALS fibroblasts following 18 h under normal conditions and under hypoxia (1% O_2._) After normalizing to cell number, secretion of VEGF protein was determined as significantly increased in hypoxic control fibroblasts compared to controls cultured under normal conditions (FC = 2.9, *P* = 0.002) (Figure 8**c**). In contrast, whilst SALS fibroblasts had a marginally higher level of VEGF at baseline compared to controls (*P* = 0.3), they failed to show a statistically significant increase following hypoxia (FC = 1.6, *P* = 0.2). Thus, the evidence indicates that the hypoxic response in SALS fibroblasts is impaired.

## Discussion

Transcriptome analysis of SALS and PLS fibroblasts was performed to establish whether dysregulated processes in the CNS are recapitulated in cells derived from peripheral tissues, such that these cells may be used as a cellular model to investigate disease pathophysiology where the genetic background of disease is unknown. Having established that the differentially expressed genes in the fibroblasts belong to biological processes previously implicated in disease pathogenesis within the CNS, comparative data analysis allowed the identification of transcripts in multiple biological pathways which are in common or specifically differentially expressed in SALS compared to PLS. The microarray results show that there is a similar percentage of genes expressed in SALS, PLS and control fibroblasts; however, there are almost twice as many genes differentially expressed in ALS fibroblasts compared to controls, than PLS fibroblasts compared to controls (SALS =416 *vs.* PLS = 209), and of those altered in PLS, 56% are also altered in ALS. This result emphasizes that whilst the two diseases are differentiated clinically by the involvement of the LMN (ALS) or not (PLS), there is considerable overlap in the pathophysiological mechanisms of disease. In addition, SALS fibroblasts demonstrate a more severe phenotype than the PLS fibroblasts, in terms of gene expression, mirroring the clinical phenotype. Categorization of the differentially expressed transcripts demonstrated that they represented genes in a wide variety of biological processes, of which the top 3 categories with the greatest number of gene expression changes, in both SALS and PLS, were transcription, signalling and metabolism. In contrast, the number of genes involved in RNA processing and the response to stress was significantly increased in SALS fibroblasts; these two biological processes have been implicated in ALS pathogenesis previously, further demonstrating the ability of peripheral tissues to recapitulate characteristics of the CNS in the ALS disease state.

### Metabolic dysfunction

Analysis of the differentially expressed metabolic genes in SALS fibroblasts suggested that there was a decrease in the availability of glucose to the cells, as suggested by the reduced expression of the insulin signalling receptors *IR* and *IGFR1*, both of which activate the glucose uptake pathway, and decreased expression of the glucose transporter *SLC2A3/GLUT3*. This is predicted to cause dysregulation of glycolysis, which would lead to a reduction in pyruvate entering the mitochondria. With the associated decreased expression in the key TCA cycle enzyme *DLST*, this would lead to diminished aerobic oxidation of energy substrates such as glucose, resulting in reduced mitochondrial oxygen consumption in SALS fibroblasts. Indeed, the consequences of the gene expression changes were supported by the functional data which showed that SALS patients had significantly reduced mitochondrial respiration compared to controls, suggestive of a reduction in oxidative phosphorylation. The SALS cases also showed reduced glycolytic capacity compared to controls, indicating that SALS fibroblasts have a reduced ability to up-regulate glycolysis in the presence of the energy deficit produced by ATP synthase inhibition, due to decreased expression of key glycolytic enzymes. An increased AMP : ATP ratio produced by the reduction in mitochondrial respiration observed in the SALS fibroblasts could not be regulated to the same degree as in control patients, due to the down-regulation in SALS fibroblasts of the *PRKAA2* subunit of AMPK, a key metabolic checkpoint regulator. Under normal circumstances, AMPK would counteract the increase in AMP : ATP ratio by increasing energy substrate catabolism. However the down-regulation of this gene in SALS patients indicates that metabolic regulation is defective in these patients, which is confirmed by the bioenergetic mitochondrial assays. Therefore, a defective glucose oxidation pathway leading to mitochondrial defects may be overcome by switching substrate energy metabolism to fatty acid oxidation. The microarray data suggest that this is indeed the case, with expression of genes controlling fatty acid synthesis being down-regulated and up-regulation of genes involved in fatty acid transport and oxidation up-regulated in the SALS patients.

Alterations in fatty acid oxidation have been observed in both animal models and ALS patients. Recent work focussing on delta-9 desaturase (SCD1), the rate-limiting enzyme in monounsaturated fatty acids synthesis has shown that SCD1 gene expression levels are reduced in SOD1 G86R mice muscle, ALS patients and by sciatic nerve crush in mice [39,40]. Knockout SCD1 mice show an increase in the gene levels of PGC1-a, PPARa and PDK4, indicating an increase in mitochondrial biogenesis and a switch from glucose to fatty acids as an energy source in the muscle [41].

It is difficult at this stage to determine what is cause and what is effect in terms of ALS metabolic dysfunction. In normal tissues the glucose-fatty acid cycle is a complex biochemical mechanism that controls fuel selection and can dynamically adapt depending on the substrate supply. Both pathways can inhibit each other, with the possibility that up-regulation of fatty acid oxidation would lead to inhibition of glucose oxidation and could potentially lead to glucose intolerance and therefore insulin resistance, which has been observed in ALS cases 42–44. However, the metabolic switching at a gene level observed in the patient fibroblasts away from aerobic glucose oxidation to fatty acid oxidation in this study may represent an inherent ‘metabolic flexibility’ to try to adapt to and compensate for the disease state, which may not be present in the CNS of ALS patients to the same extent. Increased fatty acid oxidation, either as a direct cause of the disease or a consequence of down-regulation of GLUT3 or glucose intolerance in ALS patients [43], may lead to reduced fat storage which is observed in ALS patients as disease progresses [45,46] and could lead to dyslipidemia which is a prognostic factor in the disease [47].

However, there are a number of conflicting reports surrounding dyslipidemia and its role in ALS disease progression, which may be due to differing populations, sex, inclusion criteria, study design and the heterogeneity of the disease 47–50. If SALS patients try to rely more on fatty acid oxidation to meet their energy needs, then, as observed in the G93A mouse model [51], a high fat diet may prove beneficial. Interestingly, a recent study by Dorst and colleagues showed that a high calorie dietary supplementation regime stabilizes body weight in ALS patients. However, a high carbohydrate diet produced similar results to a high fat diet [52]. Healthy adults tend to have balanced energy intake and expenditure leading to a stable body mass index (BMI) and stable energy stores [44]. This is not the case in ALS patients, where dysphagia can lead to reduced food intake and hypermetabolism of patients is often but not always observed [44,53]. This imbalance would drastically reduce fat stores, especially if cells are relying on fatty acid oxidation to meet their energy needs as suggested by our microarray data. This would lead to loss of body weight and a lower BMI, which has been observed in ALS patients, with lower BMI being associated with faster disease progression [54]. Therefore, a high calorie diet, whether carbohydrate or fat based, is suggested to be beneficial at a metabolic cellular level providing increased fuel to help patients maintain their body weight for longer.

Whilst switching to a high fat diet has been shown to be beneficial in mutant SOD1 mice by increasing survival times and hydroxybutyrate treatment in the SOD1 mice increased body weight, motor neurone counts and increased ATP synthesis in purified mitochondria [51,55]. Recent studies however have shown that pre-symptomatic mice fed the omega-3-polyunsaturated fatty acid eicosapentaenoic acid (EPA), which had previously been shown to have neuroprotective potential in rats [56,57], led to a decreased life span in G93A SOD1 mice. EPA increased 4-hydroxy-nonenal (HNE) levels in microglia and led to increased vacuolization in the dorsal and ventral horn but no loss of motor neurones [58]. Interestingly when fed at symptom onset, EPA had no significant effect on G93A SOD1 mice in terms of disease onset or survival.

These data indicate the complex nature of cellular energy metabolism, which is constantly in flux. It is still unclear how ALS affects the interplay between these pathways at a molecular level, especially in the neurone as the disease progresses, and how the disease affects the metabolic response to energy supplementation. Our work using both SOD1 [59] and SALS fibroblasts as models for ALS provide insight into how the disease affects the metabolism of peripheral tissues which share the same genetic background, show disease specific metabolic phenotypes but not the toxicity observed in the cells in the CNS. This knowledge may allow us to manipulate the energy generating pathways in the neurone and ameliorate the observed metabolic dysfunction.

### RNA processing and miRNA dysregulation

Altered RNA processing emerged as a key pathogenic mechanism in ALS, with the identification of the genes encoding the RNA binding proteins TDP-43 and FUS as causes of familial ALS and TDP-43 as a major component of the ubiquitinated protein inclusions. More recently, mutations in *hnRNPA1* have been reported in ALS [60], as well as in the RNA binding proteins TATA box binding protein associated factor 15 (*TAF15*) [61] and Ewing sarcoma breakpoint region 1 (*EWSR1*) [62], both of which belong to the same protein family as FUS. In addition, the expanded G_4_C_2_ repeat in *C9ORF72* may cause pathogenicity through an RNA toxicity mechanism, sequestering RNA binding proteins from the cytoplasm [63]. Thus, following on from the earlier literature identifying angiogenin (*ANG*) [64] and senataxin (*SETX*) [65] as RNA-related proteins which are mutated in ALS, there is now substantial evidence of dysregulated RNA processing contributing to the pathogenesis of both genetic and sporadic ALS [66,67]. Of the 28 differentially expressed genes which encode proteins related to RNA processing, all except 4 were decreased in SALS fibroblasts. Multiple RNA binding proteins, including FUS and several hnRNPs (though not hnRNPA1) were decreased, thereby providing evidence of dysregulated RNA processing occurring not only in the CNS but also in peripheral tissues of SALS patients. A potential effect of the reduction in expression of these RNA processing genes is a reduction in transcription, and of the 416 significant differentially expressed genes identified, the majority, 354 genes (85%) are decreased. Transcriptional repression has been described previously in ALS, both in a SOD1-related cell model [68] and mouse model [69]. The data presented here suggest that this is a phenomenon which can extend to sporadic disease.

Multiple components of the miRNA processing pathway were also found to be specifically differentially expressed in the SALS fibroblasts. Whilst the fold changes in gene expression were not large (range −1.52 to −2.22), they produced a significant effect on the level of multiple miRNA transcripts, with all 19 of the differentially expressed miRNAs in ALS fibroblasts showing reduced expression. Interestingly, 5 of these miRNAs, for which functions have been described, are involved in hypoxia and either regulate HIF1A or HIF1B expression or are regulated themselves by HIF1A, further supporting a dysregulation in ALS of the hypoxia response (see below).

miRNAs are stable non-protein coding RNA molecules known to post-transcriptionally regulate several target mRNA transcripts, usually by binding to the 3′UTR sequence to inhibit translation. The RNA binding proteins TDP-43 and FUS have both been shown to bind to the microprocessor complex subunit DROSHA, DICER and pri-miRNA sequences, thereby implicating miRNA in the pathophysiology of ALS [35,^70^,71]. miRNAs have also been shown to be an essential component of post-mitotic neurones, as DICER knockout in Purkinje cells led to progressive neurodegeneration [72], whilst DICER knockout specifically in post-mitotic postnatal motor neurones was associated with muscular atrophy due to loss of lumbar motor neurones, increased astrogliosis and progressive neurodegeneration.

miRNAs have been shown to be both protective and deleterious in ALS. miR-206 expression in the skeletal muscle of SOD1^G93A^ ALS mouse model was found to be increased during disease progression but knockdown exacerbated the disease, as miR-206 is thought to be involved in muscle re-innervation [73]. In contrast, a significant increase in miR-155 was seen in rat and mouse SOD1^G93A^ models as well as human ALS spinal cord. Use of anti-miR-155, however, extended survival and disease duration, though the mechanism by which this occurs has yet to be established. In contrast to the increased levels observed in the spinal cord, the SALS fibroblasts showed a decrease in miR-155 expression, reflecting perhaps a cell type specific response in the fibroblasts, as spleen from SOD1^G93A^ ALS mouse as well as monocytes from ALS patients, also show increases in miR-155 [37].

Interestingly, however, 13 of the miRNAs differentially expressed in SALS fibroblasts were also shown to be decreased in spinal cord of SALS cases. As with the fibroblast miRNA profiling, there was a significant reduction in the expression of miRNAs in the SALS spinal cord, with 239/245 significantly differentially expressed miRNAs decreased in expression, whilst only 6, (but not miRNA-155) were increased [35]. In addition, 10 of the miRNAs that were differentially expressed in the SALS fibroblasts were also decreased in a neuroblastoma knockdown model of FUS [37]; FUS expression has also been shown to be decreased in these fibroblasts. Dysregulation of miRNAs which specifically bind to TDP-43 have also been shown to be dysregulated in the cerebrospinal fluid and serum of SALS cases, as well as in immortalized lymphoblastoid cell lines from patients with mutations in *TARDBP*, *FUS*, *SOD1* and *C9ORF72* [74]. Thus, there is mounting evidence of dysregulation in miRNA expression in sporadic and genetic subtypes of ALS, as well as in other neurodegenerative disorders [75].

### Hypoxia and the response to stress

Evidence of hypoxia playing a role in the pathogenesis of ALS has been mounting following the serendipitous finding that deletion of the HIF-1 binding site in the promoter of *VEGF* of the mouse resulted in a progressive neurodegenerative phenotype reminiscent of ALS [76]. VEGF has been shown to have a neuroprotective effect on motor neurones [77], not only through the formation of micro-vessels increasing the blood supply to motor neurones, but also through a direct interaction with neuropilin-1 [76]. Subsequently, it has been shown that VEGF levels in the CSF of hypoxaemic ALS patients are decreased compared to controls [78], whilst levels of both HIF1A mRNA and protein fail to increase following hypoxia in monocytes from SALS patients [79]. The SALS fibroblasts, similar to the monocytes, also fail to exhibit increased HIF1A protein expression following hypoxia, as well as an increase in VEGF protein. Thus, there is a significant failure by ALS tissues to respond to hypoxia, but where along the hypoxia response pathway this failure lies is currently unclear.

Within the spinal cord of the SOD1^G93A^ ALS mouse model, HIF1A levels have actually been shown to increase, but most significantly in the glial cells surrounding the anterior horn motor neurones [80]. However, whilst these changes were detected at the early symptomatic stage, the subsequent hypoxia response, as measured by VEGF expression was only evident at the end stage of disease and only within the glial cells. Thus, motor neurones (in addition to fibroblasts) appear to lack the neuroprotective response to hypoxia. The lack of this response, even when the hypoxia is chronic, has been shown to have significant effects in the SOD1^G93A^ ALS mouse model. Mutant SOD1 mice which experienced chronic intermittent hypoxia showed reduced performance on the rotarod, reduced ability in the wire hanging test, poorer spatial memory as assessed by the Y-maze, and a greater loss of motor neurones, compared to the SOD1^G93A^ mice under normoxia conditions [81]. This correlates with data recorded from ALS patients, which compared nocturnal hypoxia with cognitive dysfunction [82]. The results demonstrated that patients who experience nocturnal hypoxia (as defined as the average O_2_ saturation being <95% for more than 10% of the sleep time) showed a significant decrease in their memory retention and retrieval efficiency abilities. Interestingly, the use of non-invasive ventilation has been reported to improve cognitive function of patients [83] as well as survival [84]. Therefore, it is suggested that this improved survival may be in part be due to the reduction of the patient being exposed to hypoxia, to which they are unable to mount an optimal response.

### The use of fibroblasts as a cell model

The aims of this investigation included determining whether the dysregulated biological pathways in fibroblasts were consistent with the diseased tissue in the CNS, and therefore to establish whether fibroblasts could act as a cellular model for subtypes of ALS where genetic modelling of the disease is not possible. The microarray data presented clearly shows that, particularly in SALS fibroblasts, the differentially expressed genes do correlate with biological pathways known to be dysregulated in the motor neurones and spinal cord of ALS cases. The microarray data in this report, in combination with the functional data, clearly show dysregulation of miRNA processing and the response to hypoxia as severely disrupted specifically in SALS fibroblasts, and this recapitulates pathways which have been reported as being dysregulated in the CNS of ALS cases [35,79]. Therefore, fibroblasts can provide a valuable cellular model of SALS or in instances where generation of a standard over-expression cellular model, such as the large expansions of the GGGGCC repeat in *C9ORF72* or over-expression of wild type and mutant *TARDBP*, have proven challenging.

Fibroblasts have become a commonly used cell from which to derive induced pluripotent stem cells (iPSCs) with subsequent generation of neurones and glia. Neuronal cells have been differentiated from *SOD1*, *VAPB*, *TARDBP* and *C9ORF72*-related ALS patients 85–88, and astrocytes have been differentiated from *SOD1* and *TARDBP* neuronal progenitor cells [89,90]. Whilst this strategy offers a cellular model with the genetic background of a patient within a neuronal (or glial) cell type, there are still some difficulties regarding the variability between different clones generated from the same individual, which are currently limiting their use as a robust and reliable model for gene expression analysis or high throughput drug screening. This is illustrated in a recent report where the expression levels of *TARDBP* were shown to be variable in each of 3 clones from 3 different TDP-43 mutation biosamples [91]. In contrast, fibroblasts have been used to screen a library of 2000 compounds for a compound able to rescue the mitochondrial dysfunction seen in Parkinson's disease [92]. Using *parkin*-mutant fibroblasts for the initial drug screening strategy, ursocholanic acid was shown to rescue the mitochondrial dysfunction and actually increase the activity of complexes I-IV in these fibroblasts. Further work also showed the mitochondrial respiratory chain was rescued in *LRRK2*-mutant fibroblasts, thereby widening the potential of this small molecule compound to provide some level of compensation for the mitochondrial dysfunction seen in multiple subtypes of Parkinson's disease.

In conclusion, we have established that fibroblasts can be used as a cellular model for ALS and PLS as they recapitulate previously determined dysregulated biological pathways in the CNS, and through using gene expression profiling in this study, we have provided a more detailed understanding (at the gene level) of the basis of these changes. We have demonstrated that whilst there are transcriptional changes common to both SALS and PLS in metabolic genes, there is marked transcriptional repression in the SALS fibroblasts, which is associated with an enrichment of differentially expressed genes which are involved in RNA processing and the response to stress. Therefore, we propose that fibroblasts can act as a disease model, particularly where genetic modelling is not possible or challenging, (so long as the relevant gene is expressed in this cell type), to study pathophysiological mechanisms, to identify targets amenable to therapeutic intervention and to screen for disease modifying drugs.

## Supporting Information

Table S1 Clinical details including age of disease onset and disease duration of control and patient fibroblasts.

Table S2 Q-PCR primers used for validation of gene expression changes.

Table S3 List of differentially expressed gene in SALS fibroblasts compared to controls categorized according to biological function

Table S4 List of differentially expressed gene in PLS fibroblasts compared to controls categorized according to biological function

Table S5 List of miRNAs differentially expressed in SALS fibroblasts compared to neurologically normal controls.
