# Supplementary material for: Gene expression signatures in motor neurone disease fibroblasts reveal dysregulation of metabolism, hypoxia-response and RNA processing functions
Source: Neuropathol Appl Neurobiol. 2015 Jan 29;41(2):201–26. doi: 10.1111/nan.12147 (PMC4329387; doi:10.1111/nan.12147)
Supplement: Table S1 — Clinical details including age of disease onset and disease duration of control and patient fibroblasts. [file nan0041-0201-sd1.docx]

**Supplementary Table 1:** Clinical details including age of disease onset and disease duration of control and patient fibroblasts. *patients are still alive

| **SAMPLE** | **GENDER** | **AGE AT ONSET** | **AGE AT BIOPSY** | **DISEASE DURATION** | **EXPERIMENTS** |
| --- | --- | --- | --- | --- | --- |
| **Sporadic ALS Cases** | | | | | |
| SALS018 | M | 53 | 55 | 4 | Microarray/Q-PCR/Metabolic/Hypoxia |
| SALS021 | F | 66 | 68 | 1 | Microarray/Q-PCR/Metabolic/Hypoxia |
| SALS023 | F | 70 | 71 | 2 | Microarray/Q-PCR/Metabolic/Hypoxia |
| SALS026 | F | 38 | 39 | >4* | Microarray/Q-PCR/Metabolic/Hypoxia |
| SALS027 | M | 71 | 74 | >5* | Microarray/Q-PCR/Metabolic/Hypoxia |
| SALS031 | F | 74 | 76 | 4 | Microarray/Q-PCR/Metabolic/Hypoxia |
| SALS019 | F | 58 | 60 | 2 | Q-PCR |
| SALS035 | F | 71 | 72 | 2 | Q-PCR |
| SALS037 | M | 40 | 43 | >5* | Q-PCR |
| SALS038 | M | 46 | 49 | >6* | Q-PCR |
| SALS039 | M | 42 | 45 | >5* | Q-PCR |
| **PLS Cases** | | | | | |
| PLS015 | M | 63 | 69 | >9* | Microarray |
| PLS016 | F | 70 | 78 | >11* | Microarray |
| PLS020 | F | 45 | 52 | >10* | Microarray |
| PLS022 | M | 52 | 66 | >17* | Microarray |
| PLS025 | M | 56 | 66 | >13* | Microarray |
| PLS036 | F | 49 | 66 | >20* | Microarray |
| **Control Cases** | | | | | |
| CONT002 | F | n/a | 44 | n/a | Microarray/Q-PCR/Metabolic/Hypoxia |
| CONT003 | F | n/a | 60 | n/a | Microarray/Q-PCR/Metabolic/Hypoxia |
| CONT011 | M | n/a | 54 | n/a | Microarray/Q-PCR/Metabolic/Hypoxia |
| CONT014 | M | n/a | 39 | n/a | Microarray/Q-PCR/Metabolic/Hypoxia |
| CONT001 | M | n/a | 45 | n/a | Microarray/Q-PCR/Hypoxia |
| CONT013 | F | n/a | 53 | n/a | Microarray/Q-PCR/Hypoxia |
| CONT004 | M | n/a | 50 | n/a | Q-PCR |
| CONT006 | F | n/a | 56 | n/a | Q-PCR |
| CONT009 | M | n/a | 39 | n/a | Q-PCR |
| CONT012 | M | n/a | 44 | n/a | Q-PCR |
| CONT005 | F | n/a | 54 | n/a | Metabolic |
| CONT008 | F | n/a | 37 | n/a | Metabolic |
| CONT019 | F | n/a | 79 | n/a | Metabolic |
| CONT156 | F | n/a | 74 | n/a | Metabolic |
| CONT157 | F | n/a | 76 | n/a | Metabolic |
|  |  |  |  |  |  |
