# Supplementary material for: Gene expression signatures in motor neurone disease fibroblasts reveal dysregulation of metabolism, hypoxia-response and RNA processing functions
Source: Neuropathol Appl Neurobiol. 2015 Jan 29;41(2):201–26. doi: 10.1111/nan.12147 (PMC4329387; doi:10.1111/nan.12147)
Supplement: Table S2 — Q-PCR primers used for validation of gene expression changes. [file nan0041-0201-sd2.docx]

**Supplementary Table 2:** Real-time PCR primer sequences and optimised concentrations for Q-PCR

| **Gene Symbol** | **Primer Sequences** | **Concentration** |
| --- | --- | --- |
| ACTB | F 5’ GAGCTACGAGCTGCCTGACG 3’ | 300nM |
|  | R 5’GTAGTTTCGTGGATGCCACAG 3’ | 300nM |
| AGO1/EIF2C1 | F 5’ CAAGCCCCAGGAGCTGTGCC 3’ | 300nM |
|  | R 5’ GCTGGCCCTGTTCCCCACTC 3’ | 300nM |
| ARNT | F 5’ TCACATTGCACGCCTCCCCCCT 3’ | 300nM |
|  | R 5’ AACGCCCACCCCAAAACCCC 3’ | 300nM |
| ARNT2 | F 5’ CCAGGGTCTGTGGGGCACTG 3’ | 300nM |
|  | F 5’ AAGCAAGCCGTCCTGCTCCC 3’ | 300nM |
| DICER1 | F 5' TACCCCGTTCCCCTGTGCGA 3' | 300nM |
|  | R 5' CCCGTCGTAAGTTCTCTCAGCCG 3' | 300nM |
| FAC1 | F 5’ AATCGGAGAAGTCCAACGGGGAG 3’  R 5’ GCTGCCACCTGCTGGCTCTT 3’ | 300nM  300nM |
| FADS2 | F 5’ TTTCGGGGAGGGCGCCTAGT 3’ | 300nM |
|  | R 5’ TGCAGGTCCCTCCTGGCTGC 3’ | 300nM |
| FUS | F 5’ AAACTTTTTGGGATCTGAGTCCTTT 3’ | 300nM |
|  | R 5’ AGCTACTGAGGAGGCTGAGGC 3’ | 300nM |
| SLC2A3/GLUT3 | F 5’ GCTCACGGCACGCTTGCGTA 3’ | 300nM |
|  | R 5’ GTCCCCTGAGGGCATTCGGC 3’ | 300nM |
| HIF-1A | F 5’ AGCATGTAGACTGCTGGGGCAA 3’ | 300nM |
|  | R 5’ CCTGCAGTAGGTTTCTGCTGCCTTG 3’ | 300nM |
| IGF1R | F 5’CCTCGGCCAGGAATCCA 3’ | 300nM |
|  | R 5’AAGCAACTCTTGTGCTGTCATCTC 3’ | 300nM |
| PCBP2/HNRNPE2 | F 5' CGGGCGTCAAGGCGCCAAAA 3' | 300nM |
|  | R 5' CTGCGCCCCAGACATCTGACG 3' | 300nM |
| PGK1 | F 5’ GGTGGTGGAATGGCTTTTACC 3’ | 300nM |
|  | R 5’ TCCACTTGGCATTTGTTTCCT 3’ | 300nM |
| PRKAA2 | F 5’GGTGACAGGCTGCTTGTAATCC 3’ | 300nM |
|  | R 5’TCTGCCTCCCAGGTTCAAGA 3’ | 300nM |
| SLC27A4/FATP4 | F 5’ GGCTGAGACTGACGGGTTTTC 3’ | 900nM |
|  | R 5’ GGCTCGGTGACCCCTTGT 3’ | 300nM |
| VEGF | F 5’ TACCTCCACCATGCCAAGTG 3’ | 300nM |
|  | R 5’ GATGATTCTGCCCTCCTCCTT 3’ | 300nM |
