# Supplementary material for: Gene expression signatures in motor neurone disease fibroblasts reveal dysregulation of metabolism, hypoxia-response and RNA processing functions
Source: Neuropathol Appl Neurobiol. 2015 Jan 29;41(2):201–26. doi: 10.1111/nan.12147 (PMC4329387; doi:10.1111/nan.12147)
Supplement: Table S3 — List of differentially expressed gene in SALS fibroblasts compared to controls categorized according to biological function [file nan0041-0201-sd3.docx]

**Supplementary Table 3:** List of differentially expressed gene in SALS fibroblasts compared to controls categorised according to biological function.

| **Probe Set ID** | **Gene Symbol** | **Gene Name** | **Regulation** | **Fold Change** | **p-value** | **Altered in PLS?** |
| --- | --- | --- | --- | --- | --- | --- |
| **Apoptosis and Cell Survival** | | | | | | |
| 210334_x_at | BIRC5 | baculoviral IAP repeat-containing 5 | up | 1.6555 | 0.0203 | PLS |
| 241722_x_at | MCL1 | myeloid cell leukemia sequence 1 (BCL2-related) | down | 1.6364 | 0.0204 |  |
| 1568763_s_at | PDCD6 | programmed cell death 6 | down | 1.6732 | 0.0434 |  |
| 217999_s_at | PHLDA1 | pleckstrin homology-like domain, family A, member 1 | up | 1.6023 | 0.0136 | PLS |
| 218424_s_at | STEAP3 | STEAP family member 3 | up | 1.5246 | 0.0010 |  |
| **Cell Adhesion** | | | | | | |
| 228573_at | ANTXR2 | anthrax toxin receptor 2 | down | 1.5662 | 0.0143 |  |
| 237310_at | EXT1 | exostosin (multiple)1 | down | 1.8432 | 0.0143 |  |
| 236251_at | ITGAV | integrin alpha V | down | 1.9555 | 0.0075 |  |
| 219213_at | JAM2 | junctional adhesion molecule 2 | down | 2.1946 | 0.0015 | PLS |
| 208933_s_at | LGALS8 | lectin, galactoside-binding, soluble, 8 | down | 1.6037 | 0.0055 | PLS |
| 204580_at | MMP12 | matrix metalloproteinase 12 | up | 5.1085 | 0.0110 | PLS |
| 205828_at | MMP3 | matrix metalloproteinase 3 | up | 3.4764 | 0.0218 | PLS |
| 214375_at | PPFIBP1 | similar to PTPRF interacting protein binding protein 1 | down | 1.5094 | 0.0250 |  |
| 229465_s_at | PTPRS | protein tyrosine phosphatase, receptor type, sigma | down | 2.0559 | 0.0407 |  |
| 1558115_at | RECK | RECK protein | down | 1.5896 | 0.0074 |  |
| **Cell Cycle** | | | | | | |
| 232266_x_at | CDC2L5 | CDC2-related protein kinase 5 | down | 1.5547 | 0.0268 |  |
| 1563223_a_at | CENPI | centromere protein 1 | down | 1.9495 | 0.0059 |  |
| 226611_s_at | CENPV | centromere protein V | down | 1.8390 | 0.0062 | PLS |
| 205022_s_at | FOXN3 | forkhead box N3 | down | 1.7149 | 0.0139 | PLS |
| 223380_s_at | LATS2 | LATS (large tumor suppressor, Drosophila) homolog 2 | down | 1.6033 | 0.0495 |  |
| 223189_x_at | MLL5 | myeloid/lymphoid or mixed-lineage leukemia 5 | down | 1.8989 | 0.0323 |  |
| 213328_at | NEK1 | NIMA-related kinase 1 | down | 1.5764 | 0.0383 |  |
| 215462_at | PLK3 | polo-like kinase 3 (Drosophila) | down | 1.5198 | 0.0016 |  |
| 238040_at | POGZ | pogo transposable element with ZNF domain | down | 2.6220 | 0.0188 |  |
| 1559038_at | SEPT2 | septin-2 | down | 1.5349 | 0.0203 |  |
| 212413_at | SEPT6 | septin 6 | down | 1.5741 | 0.0120 | PLS |
| 227862_at | TRNP1 | TMF regulated nuclear protein | up | 1.6130 | 0.0049 |  |
| **Cytoskeleton** | | | | | | |
| 232058_at | ACTN4 | actinin alpha 4 | down | 1.5742 | 0.0011 |  |
| 222024_s_at | AKAP13 | A kinase (PRKA) anchor protein 13 | down | 1.6918 | 0.0174 |  |
| 228993_s_at | BBIP1 | BBS protein complex-interacting protein | down | 2.5407 | 0.0393 |  |
| 235834_at | CALD1 | caldesmon 1 | down | 2.4293 | 0.0262 |  |
| 1557810_at | CCT5 | chaperonin containing TCP1, subunit 5 (epsilon) | down | 1.5981 | 0.0364 |  |
| 227850_x_at | CDC42EP5 | CDC42 effector protein 5 | down | 1.6784 | 0.0215 |  |
| 210762_s_at | DLC1 | deleted in liver cancer 1 | down | 1.6619 | 0.0277 |  |
| 205493_s_at | DPYSL4 | dihydropyrimidinase-like 4 | down | 3.1022 | 0.0103 | PLS |
| 206124_s_at | LLGL1 | lethal giant larvae homolog 1 | up | 1.5921 | 0.0025 |  |
| 55065_at | MARK4 | MAP/microtubule affinity-regulating kinase 4 | down | 1.5306 | 0.0005 |  |
| 200907_s_at | PALLD | palladin, cytoskeletal associated protein | down | 1.5658 | 0.0427 |  |
| 225548_at | SHROOM3 | shroom-related protein | down | 1.7584 | 0.0402 |  |
| 242220_at | SPTBN1 | spectrin, beta, non-erythrocytic 1 | up | 1.6082 | 0.0353 |  |
| 213201_s_at | TNNT1 | troponin-T1, skeletal, slow | down | 1.7494 | 0.0062 | PLS |
| **Immune response** | | | | | | |
| 223710_at | CCL26 | chemokine (C-C motif) ligand 26 | up | 1.6327 | 0.0021 | PLS |
| 224991_at | CMIP | c-Maf-inducing protein | down | 1.7091 | 0.0137 |  |
| 225009_at | CMTM4 | CKLF-like MARVEL transmembrane domain containing 4 | down | 1.8725 | 0.0162 |  |
| 1565674_at | FCGR2A/B/C | Fc fragment of IgG, low affinity IIa, receptor (CD32) | up | 1.5655 | 0.0069 |  |
| 214022_s_at | IFITM1 | Interferon-induced trans membrane protein 1 | down | 1.8617 | 0.0143 | PLS |
| 211632_at | IGHD | immunoglobulin heavy constant delta | down | 2.0868 | 0.0017 | PLS |
| 205904_at | MICA | MHC class I polypeptide-related sequence A | up | 1.6354 | 0.0042 | PLS |
| **Ion Transport** | | | | | | |
| 207103_at | KCND2 | potassium voltage-gated channel, Shal-related subfamily, member 2 | down | 1.7416 | 0.0149 | PLS |
| 235857_at | KCTD11 | potassium channel tetramerisation domain containing 11 | down | 1.8229 | 0.0215 | PLS |
| 223176_at | KCTD20 | potassium channel tetramerisation domain containing 20 | down | 2.0092 | 0.0086 | PLS |
| 220791_x_at | SCN11A | sodium channel, voltage-gated, type XI, alpha | down | 1.5831 | 0.0317 |  |
| 232055_at | SFXN1 | sideroflexin 1 | down | 1.8674 | 0.0175 |  |
| 241941_at | ACSBG2 | acyl-CoA synthetase bubblegum family member 2 | down | 1.5806 | 0.0048 |  |
| **Metabolism** | | | | | | |
| 226783_at | AGXT2L2 | alanine-glyoxylate aminotransferase 2-like 2 | down | 1.5109 | 0.0274 |  |
| 202587_s_at | AK1 | Adenylate kinase 1 | up | 1.6461 | 0.0001 | PLS |
| 230630_at | AK4/AK3L1 | adenylate kinase 4/adenylate kinase 3 like 1 | down | 1.5859 | 0.0155 | PLS |
| 229403_at | B4GALT1 | UDP-Gal:beta-GlcNAc beta-1,4-galactosyltransferase 1 | down | 2.4104 | 0.0010 |  |
| 215210_s_at | DLST | dihydrolipoamide S-succinyltransferase (E2 componenet of oxo-glutarate complex) | down | 1.5120 | 0.0042 |  |
| 213787_s_at | EBP | emopamil binding protein (sterol isomerase) | up | 1.7422 | 0.0053 | PLS |
| 202218_s_at | FADS2 | fatty acid desaturase 2 | down | 3.2331 | 0.0003 | PLS |
| 224889_at | FOXO3 | forkhead box O3 | down | 1.6720 | 0.0014 |  |
| 203628_at | IGF1R | insulin like growth factor-1 receptor | down | 2.5562 | 0.0401 |  |
| 227377_at | IGF2BP1 | insulin-like growth factor 2 mRNA binding protein 1 | up | 3.8345 | 0.0034 | PLS |
| 218847_at | IGF2BP2 | insulin-like growth factor 2 mRNA-binding protein 2 | down | 1.6330 | 0.0312 |  |
| 227432_s_at | IR | insulin receptor | down | 3.0820 | 0.0120 | PLS |
| 211752_s_at | NDUFS7 | NADH-coenzyme Q reductase | up | 1.5355 | 0.0083 |  |
| 203189_s_at | NDUFS8 | NADH dehydrogenase ubiquinone Fe-S 8 | up | 1.6898 | 0.0021 |  |
| 201577_at | NME1/NDPKA | non-metastatic cells 1/ nucleoside disphosphate kinase A | up | 1.6117 | 0.0018 | PLS |
| 201695_s_at | NP | purine nucleoside phosphorylase | up | 1.5636 | 0.0417 |  |
| 230109_at | PDE7B | phosphodiesterase 7B | down | 1.6006 | 0.0279 | PLS |
| 1558365_at | PGK1 | phosphoglycerate kinase 1 | down | 1.7449 | 0.0343 |  |
| 229553_at | PGM2L1 | phosphoglucomutase 2-like 1 | down | 1.6171 | 0.0101 | PLS |
| 1559496_at | PPA2 | pyrophosphatase (inorganic) 2 | down | 1.5233 | 0.0368 |  |
| 227892_at | PRKAA2 | AMP-activated protein kinase alpha 2 catalytic subunit | down | 1.5513 | 0.0139 |  |
| 230352_at | PRPS2 | phosphoribosyl pyrophosphate synthetase 2 | down | 3.0996 | 0.0463 |  |
| 243543_at | SC4MOL | sterol-C4-methyl-oxidase like | down | 1.5576 | 0.0397 |  |
| 200832_s_at | SCD | stearoyl-CoA desaturase (delta-9-desaturase) | down | 1.7845 | 0.0264 |  |
| 220232_at | SCD5 | stearoyl-CoA desaturase 5 | down | 1.9712 | 0.0071 | PLS |
| 201917_s_at | SLC25A36 | solute carrier family 25, member 36 | down | 1.7157 | 0.0025 | PLS |
| 231274_s_at | SLC25A37 | solute carrier family 25, member 37 | down | 2.4865 | 0.0263 |  |
| 225779_at | SLC27A4/FATP4 | solute carrier family 27 (fatty acid transporter), member 4 | up | 1.5658 | 0.0064 | PLS |
| 202499_s_at | SLC2A3/GLUT3 | solute carrier family 2 (facilitated glucose transporter), member 3 | down | 1.6341 | 0.0027 | PLS |
| **Miscellaneous** | | | | | | |
| 220016_at | AHNAK | AHNAK nucleoprotein | down | 1.5931 | 0.0019 |  |
| 214707_x_at | ALMS1 | Alstrom syndrome 1 | down | 1.5671 | 0.0359 |  |
| 225852_at | ANKRD17 | ankyrin repeat domain 17 | down | 1.5536 | 0.0323 |  |
| 220874_at | ASTN2 | astrotactin 2 | down | 1.6619 | 0.0435 |  |
| 242693_at | CDK13 | cyclin dependent kinase 13 | down | 1.8680 | 0.0043 | PLS |
| 229171_at | CENPBD1 | CENPB DNA-binding domains containing 1 | down | 1.6684 | 0.0222 | PLS |
| 1554241_at | COCH | coagulation factor C (Limulus polyphemus homolog); cochlin | down | 1.8882 | 0.0316 | PLS |
| 1559332_at | CRIM1 | cysteine rich transmembrane BMP regulator 1 (chordin-like) | down | 1.5997 | 0.0466 |  |
| 209674_at | CRY1 | cryptochrome 1 (photolyase-like) | down | 1.8498 | 0.0293 |  |
| 216778_s_at | CYLC1 | Cylicin I | up | 1.6150 | 0.0280 |  |
| 207896_s_at | DLEC1 | Deleted in lung cancer protein 1 | down | 2.0259 | 0.0037 |  |
| 215252_at | DNAJC7 | DnaJ (Hsp40) homolog, subfamily C, member 7 | down | 1.7258 | 0.0116 | PLS |
| 203729_at | EMP3 | epithelial membrane protein 3 | up | 1.5611 | 0.0135 |  |
| 208503_s_at | GATAD1 | GATA zinc finger domain containing 1 | down | 1.6153 | 0.0123 | PLS |
| 235371_at | GLT8D4 | glycosyltransferase 8 domain containing 4 | down | 1.9991 | 0.0093 | PLS |
| 217631_at | GTPBP4 | GTP binding protein 4 | down | 1.5136 | 0.0227 |  |
| 45714_at | HCFC1R1 | host cell factor C1 regulator 1 | down | 1.5164 | 0.0415 |  |
| 229400_at | HOXD10 | homeo box D10 | down | 1.6783 | 0.0137 | PLS |
| 1560999_a_at | IL12RB2 | interleukin 12 receptor, beta 2 | down | 1.9505 | 0.0029 |  |
| 229638_at | IRX3 | iroquois homeobox 3 | down | 1.8433 | 0.0213 |  |
| 238119_at | MFF | mitochondrial fission factor | down | 2.0254 | 0.0100 | PLS |
| 225954_s_at | MIDN | Midbrain nucleolar protein | down | 2.0386 | 0.0174 |  |
| 203345_s_at | MTF2 | Metal regulatory transcription factor 2 | down | 1.5057 | 0.0468 |  |
| 1569617_at | OSBP2 | oxysterol binding protein 2 | down | 1.9900 | 0.0005 |  |
| 211496_s_at | PDC | phosducin | down | 1.8426 | 0.0054 | PLS |
| 244826_at | PITPNB | phosphatidylinositol transfer protein, beta | down | 1.7453 | 0.0079 |  |
| 218901_at | PLSCR4 | phospholipid scramblase 4 | down | 1.6386 | 0.0015 | PLS |
| 222653_at | PNPO | pyridoxine 5'-phosphate oxidase | up | 1.6345 | 0.0013 | PLS |
| 227192_at | PRRT2 | proline-rich transmembrane protein 2 | down | 1.5907 | 0.0093 |  |
| 219550_at | ROBO3 | Roundabout-like protein 3 | down | 1.6061 | 0.0148 |  |
| 234491_s_at | SAV1 | salvador homolog 1 | down | 1.5246 | 0.0007 |  |
| 217811_at | SELT | selenoprotein T | up | 1.5390 | 0.0002 |  |
| 209723_at | SERPINB9 | serpin peptidase inhibitor, clade B (ovalbumin), member 9 | down | 1.6001 | 0.0238 | PLS |
| 1553089_a_at | WFDC2 | WAP four-disulfide core domain 2 | up | 1.5070 | 0.0092 |  |
| 1559515_at | XPA | xeroderma pigmentosum, complementation group A | down | 2.0953 | 0.0021 | PLS |
| **Protein Catabolism** | | | | | | |
| 235926_at | ANAP5 | anaphase promoting complex subunit 5 | down | 1.7302 | 0.0439 |  |
| 1553682_at | FBXL14 | F-box and leucine-rich repeat protein 14 | down | 1.5897 | 0.0073 |  |
| 1555413_s_at | FBXL21 | F-box and leucine-rich repeat protein 21 | up | 1.5227 | 0.0170 |  |
| 232693_s_at | FBXO16 | F-box protein 16 | down | 1.5533 | 0.0281 |  |
| 212229_s_at | FBXO21 | F-box protein 21 | down | 1.5241 | 0.0120 |  |
| 1563659_at | HERC6 | HECT and RLD domain containing E3 ubiquitin protein ligase family member 6 | down | 1.6904 | 0.0016 |  |
| 236235_at | ITCH | itchy E3 ubiquitin protein ligase homolog (mouse) | down | 1.8713 | 0.0189 | PLS |
| 233819_s_at | LTN1 | listerin E3 ubiquitin protein ligase 1 | down | 1.6866 | 0.0187 | PLS |
| 201737_s_at | MARCH6 | Membrane associated ring finger protein (CC3HC4)6 | down | 1.9120 | 0.0395 |  |
| 202243_s_at | PSMB4 | proteasome beta 4 subunit | up | 1.6609 | 0.0303 |  |
| 201267_s_at | PSMC3 | Proteasome 26S subunit ATPase 3 | up | 1.5137 | 0.0423 |  |
| 201011_at | RPN1 | Ribophorin-1 | up | 1.5400 | 0.0207 |  |
| 238391_at | SMURF2 | SMAD specific E3 ubiquitin protein ligase 2 | down | 1.8035 | 0.0027 |  |
| 223014_at | UBE2R2 | ubiquitin-conjugating enzyme E2R 2 | down | 1.5294 | 0.0142 |  |
| 201296_s_at | WSB1 | WD repeat and SOCS box containing 1 | down | 1.7314 | 0.0012 | PLS |
| **Protein Processing** | | | | | | |
| 229585_at | ADAMTSL1 | ADAMTS-like 1 | down | 1.5489 | 0.0269 |  |
| 208994_s_at | PPIG | peptidylprolyl isomerase G (cyclophilin G) | down | 1.5414 | 0.0258 |  |
| **Protein Translation** | | | | | | |
| 212225_at | EIF1 | eukaryotic translation initiation factor 1 | up | 1.8200 | 0.0024 |  |
| 214314_s_at | EIF5B | eukaryotic translation initiation factor 5B | down | 1.5623 | 0.0257 |  |
| 1556272_a_at | NARS2 | asparaginyl-tRNA synthetase 2, mitochondrial (putative) | down | 1.9920 | 0.0036 |  |
| **Protein Transport** | | | | | | |
| 1556931_at | CDC42 | cell division cycle 42 (GTP binding protein, 25kDa); cell division cycle 42 pseudogene 2 | down | 1.5155 | 0.0102 |  |
| 242629_at | Rab3b | RAB3B, member RAS oncogene family | down | 2.3953 | 0.0324 |  |
| 202975_s_at | RHOBTB3 | Rho-related BTB domain containing 3 | down | 1.9099 | 0.0053 | PLS |
| 238909_at | S100A10 | S100 calcium-binding protein A10 | down | 1.5682 | 0.0068 |  |
| 242160_at | SNTX17 | syntaxin 17 | down | 1.5964 | 0.0307 |  |
| 217960_s_at | TOMM22 | Translocase of outer membrane 22 kDa subunit homolog | up | 1.6900 | 0.0089 | PLS |
| 201730_s_at | TPR | translocated promoter region (to activated MET oncogene) | down | 1.9726 | 0.0349 |  |
| **Response to Stress** | | | | | | |
| 210558_at | AKR1C4 | aldo-keto reductase family 1, member C4 | up | 1.7551 | 0.0238 |  |
| 231016_s_at | ARNT/HIF1B | aryl hydrocarbon receptor nuclear translocator | down | 2.4090 | 0.0174 | PLS |
| 202986_at | ARNT2 | aryl hydrocarbon receptor nuclear translocator 2 | down | 1.7389 | 0.0078 | PLS |
| 229456_s_at | DDAH1 | dimethylarginine dimethylaminohydrolase 1 | down | 3.2078 | 0.0205 |  |
| 201041_s_at | DUSP1 | dual specificity phosphatase 1 | up | 1.8165 | 0.0284 |  |
| 202221_s_at | EP300 | histone acetyltransferase p300 | down | 1.8696 | 0.0259 |  |
| 207186_s_at | FAC1 | fetal Alzheimer antigen | down | 2.0544 | 0.0095 |  |
| 209189_at | FOS | v-fos FBJ murine osteosarcoma viral oncogene homolog | up | 2.4329 | 0.0045 |  |
| 200736_s_at | GPX1 | glutathione peroxidase 1 | up | 1.5705 | 0.0391 |  |
| 238869_at | HIF1A | hypoxia inducible factor 1A | down | 1.6450 | 0.0319 |  |
| 214246_x_at | MINK1 | misshapen-like kinase 1 | down | 1.5812 | 0.0040 |  |
| 213791_at | PENK | enkephalin A | up | 4.2434 | 0.0116 |  |
| 212783_at | RBBP6 | retinoblastoma-binding protein 6 | down | 1.6002 | 0.0002 |  |
| 223519_at | ZAK | leucine zipper sterile alpha motif containing kinase | down | 2.0154 | 0.0382 |  |
| **RNA Processing** | | | | | | |
| 222576_s_at | AGO1/EIF2C1 | Argonaute 1/ eukaryotic translation initiation factor 2C 1 | down | 1.5460 | 0.0403 |  |
| 229841_at | AGO2/EIF2C2 | Argonaute 2/ eukaryotic translation initiation factor 2C 2 | down | 2.2779 | 0.0111 | PLS |
| 238913_at | CPSF6 | cleavage and polyadenylation specific factor 6 | down | 1.5832 | 0.0016 |  |
| 64474_g_at | DGCR8 | DiGeorge syndrome critical region 8 | down | 1.5243 | 0.0211 |  |
| 206061_s_at | DICER1 | Dicer1, Dcr-1 homolog | down | 2.0345 | 0.0366 |  |
| 218695_at | EXOSC4 | exosome component 4 | up | 1.5284 | 0.0037 |  |
| 231108_at | FUS | Fused in liposarcoma | down | 1.7869 | 0.0176 |  |
| 201514_s_at | G3BP1 | GTPase activating protein (SH3 domain) binding protein 1 | up | 1.5249 | 0.0235 | PLS |
| 235999_at | HNRNPD | heterogeneous nuclear ribonucleoprotein D | down | 1.6409 | 0.0065 |  |
| 238611_at | HNRNPM | heterogeneous nuclear ribonucleoprotein M | down | 1.6457 | 0.0237 |  |
| 236244_at | HNRNPU | heterogeneous nuclear ribonucleoprotein U | down | 1.8483 | 0.0313 |  |
| 227555_s_at | KHSRP | FUSE-binding protein 2 | down | 1.9355 | 0.0189 |  |
| 202736_s_at | LSM4 | U6 snRNA-associated Sm-like protein 4 | up | 1.5242 | 0.0319 |  |
| 235879_at | MBNL1 | muscleblind-like 1 | down | 1.9694 | 0.0117 |  |
| 220110_s_at | NXF3 | nuclear RNA export factor 3 | down | 1.5791 | 0.0214 |  |
| 213517_at | PCBP2/ HNRNPE2 | poly(rC) binding protein 2/ heterogeneous nuclear ribonucleoprotein E2 | down | 2.1358 | 0.0394 |  |
| 219408_at | PRMT7 | protein arginine methyltransferase 7 | up | 1.5407 | 0.0113 |  |
| 212263_at | QKI | quaking homolog, KH domain RNA binding (mouse) | down | 1.5299 | 0.0113 |  |
| 207836_s_at | RBPMS | RNA-binding protein with multiple splicing | down | 1.5708 | 0.0039 |  |
| 236621_at | RPS27 | ribosomal protein S27 pseudogene | down | 1.5346 | 0.0106 |  |
| 212177_at | SFRS18 | splicing factor, arginine/serine-rich 18 | down | 1.6220 | 0.0059 | PLS |
| 242146_at | SNRPA1 | small nuclear ribonucleoprotein polypeptide A | down | 1.7213 | 0.0204 |  |
| 227891_s_at | TAF15 | TAF15 RNA polymerase II, TATA box binding protein | down | 1.6525 | 0.0360 |  |
| 228483_s_at | TAF9B | TAF9B RNA polymerase II, TATA box binding protein (TBP)-associated factor | down | 2.3297 | 0.0060 | PLS |
| 235716_at | TRA2A | transformer 2 alpha homolog (Drosophila) | down | 2.5670 | 0.0174 |  |
| 1557300_s_at | WHSC2 | Wolf-Hirschhorn syndrome candidate 2 | down | 1.5367 | 0.0126 |  |
| 227621_at | WTAP | Wilms tumor 1 associated protein | down | 2.1900 | 0.0481 |  |
| 201369_s_at | ZFP36L2 | zinc finger protein 36, C3H type-like 2 | down | 1.6478 | 0.0485 |  |
| **Signalling** | | | | | | |
| 229307_at | ANKRD28 | ankyrin repeat domain 28 | down | 2.3366 | 0.0251 | PLS |
| 205068_s_at | ARHGAP26 | Rho GTPase activating protein 26 | down | 1.5514 | 0.0338 |  |
| 1554703_at | ARHGEF10 | Rho guanine nucleotide exchange factor 10 | down | 1.7450 | 0.0002 |  |
| 242036_x_at | ATP2B3 | ATPase, Ca++ transporting, plasma membrane 3 | down | 1.9042 | 0.0039 |  |
| 1556007_s_at | CSNK1A1 | casein kinase 1, alpha 1 | down | 2.0616 | 0.0063 |  |
| 213979_s_at | CTBP1 | C-terminal binding protein 1 | down | 3.9639 | 0.0464 |  |
| 1555989_at | DAAM1 | dishevelled associated activator of morphogenesis 1 | down | 4.2847 | 0.0020 |  |
| 201280_s_at | DAB2 | disabled homolog 2 | down | 1.8024 | 0.0220 |  |
| 211235_s_at | ESR1 | estrogen receptor 1 | up | 1.5112 | 0.0283 |  |
| 214240_at | GAL | galanin prepropeptide | up | 2.1423 | 0.0016 | PLS |
| 220821_at | GALR1 | galanin receptor 1 | down | 1.5458 | 0.0226 |  |
| 235851_s_at | GNAS | guanine nucleotide binding protein (G protein), alpha | down | 1.8754 | 0.0093 | PLS |
| 223487_x_at | GNB4 | G protein beta-4 subunit | down | 2.1130 | 0.0259 |  |
| 65718_at | GPR124 | G protein-coupled receptor 124 | down | 1.7490 | 0.0046 | PLS |
| 217641_at | GPR135 | G protein-coupled receptor 135 | down | 1.5715 | 0.0015 |  |
| 1559688_at | GRAPL | GRB2-related adaptor protein-like | down | 1.8844 | 0.0170 |  |
| 215659_at | GSDMB | gasdermin B | down | 1.5158 | 0.0369 |  |
| 1565627_a_at | LRRK1 | leucine-rich repeat kinase 1 | down | 1.9168 | 0.0415 |  |
| 238761_at | MED28 | mediator complex subunit 28 | down | 1.9452 | 0.0330 |  |
| 211599_x_at | MET | met proto-oncogene (hepatocyte growth factor receptor) | up | 1.7572 | 0.0279 |  |
| 1558220_at | MUC20 | mucin 20, cell surface associated | down | 1.7658 | 0.0080 | PLS |
| 219789_at | NPR3 | natriuretic peptide receptor C/guanylate cyclase C | down | 2.4427 | 0.0015 | PLS |
| 228014_at | PLXNA4 | plexin A4 | up | 1.5294 | 0.0023 | PLS |
| 236754_at | PPP1R2 | protein phosphatase 1, regulatory (inhibitor) subunit 2 | down | 1.5539 | 0.0006 |  |
| 204284_at | PPP1R3C | protein phosphatase 1, regulatory (inhibitor) subunit 3C | up | 1.5258 | 0.0115 |  |
| 209815_at | PTCH1 | patched homolog 1 (Drosophila) | down | 1.6651 | 0.0006 | PLS |
| 200636_s_at | PTPRF | protein tyrosine phosphatase, receptor type, F | down | 1.5475 | 0.0069 |  |
| 202844_s_at | RALBP1 | Ral-interacting protein 1 | down | 1.6393 | 0.0465 |  |
| 230669_at | RASA2 | RAS p21 protein activator 2 | down | 1.7002 | 0.0459 |  |
| 244055_at | RYR3 | ryanodine receptor 3 | down | 1.5103 | 0.0394 |  |
| 41644_at | SASH1 | SAM and SH3 domain containing 1 | down | 1.8342 | 0.0103 |  |
| 1560889_a_at | SEMA5A | sema domain, seven thrombospondin repeats (type 1 and type 1-like), transmembrane domain (TM) and short cytoplasmic domain, (semaphorin) 5A | down | 1.9224 | 0.0025 |  |
| 205405_at | SEMA5A | semaphorin 5A | down | 2.0752 | 0.0249 |  |
| 1568765_at | SERPINE1 | serpin peptidase inhibitor, clade E (nexin, plasminogen activator inhibitor type 1), member 1 | down | 2.2946 | 0.0215 |  |
| 201810_s_at | SH3BP5 | SH3 binding protein 5 | down | 1.9761 | 0.0112 |  |
| 225227_at | SKIL | SKI-like oncogene | down | 1.5140 | 0.0415 |  |
| 221016_s_at | TCF7L1 | transcription factor 7-like 1 | down | 1.8427 | 0.0010 | PLS |
| 212762_s_at | TCF7L2 | transcription factor 7-like 2 | down | 2.1988 | 0.0017 | PLS |
| 205016_at | TGFA | transforming growth factor-alpha | up | 2.2162 | 0.0283 |  |
| 222736_s_at | TMEM38B | transmembrane protein 38B | up | 1.7242 | 0.0038 | PLS |
| 231227_at | WNT5A | wingless-type MMTV integration site family, member 5A | down | 2.3938 | 0.0328 |  |
| **Transcription** | | | | | | |
| 219437_s_at | ANKRD11 | ankyrin repeat domain 11 | down | 1.8524 | 0.0138 | PLS |
| 231090_s_at | ARID2 | AT rich interactive domain 2 (ARID, RFX-like) | down | 1.9847 | 0.0229 | PLS |
| 222667_s_at | ASH1L | ASH1-like protein | down | 1.6924 | 0.0387 |  |
| 204999_s_at | ATF5 | activating transcription factor 5 | down | 1.8299 | 0.0439 |  |
| 236778_at | ATRX | alpha thalassemia/mental retardation syndrome X-linked (RAD54 homolog, S. cerevisiae) | down | 1.5263 | 0.0301 |  |
| 204908_s_at | BCL3 | B-cell CLL/lymphoma 3 | down | 1.6383 | 0.0078 | PLS |
| 223915_at | BCOR | BCL-6 interacting corepressor | down | 1.6490 | 0.0120 |  |
| 230368_at | ERF | Ets2 repressor factor | down | 1.8669 | 0.0009 |  |
| 239364_at | Etv6 | ets variant 6 | down | 2.0733 | 0.0080 | PLS |
| 241824_at | FOSL2 | FOS-like antigen 2 | down | 1.5937 | 0.0374 |  |
| 224837_at | FOXP1 | forkhead box P1 | down | 1.5271 | 0.0078 | PLS |
| 225393_at | GATAD2B | GATA zinc finger domain containing 2B | down | 1.5601 | 0.0468 |  |
| 212966_at | HIC2 | hypermethylated in cancer 2 | down | 1.8013 | 0.0014 |  |
| 208546_x_at | HIST1H2BH | histone 1, H2bh | up | 1.5575 | 0.0130 | PLS |
| 208180_s_at | HIST1H4B | histone 1, H24B | up | 1.5035 | 0.0056 |  |
| 1562236_at | KAT6B | K(lysine) acetyltransferase 6B | down | 2.3865 | 0.0050 |  |
| 208987_s_at | KDM2A | lysine-specific demethylase 2A | down | 1.9410 | 0.0458 |  |
| 203542_s_at | KLF9 | Kruppel-like factor 9 | down | 2.0181 | 0.0023 | PLS |
| 236241_at | MED31 | mediator complex subunit 31 | down | 1.6395 | 0.0006 | PLS |
| 202364_at | MXI1 | MAX interactor 1 | down | 1.5333 | 0.0097 | PLS |
| 214656_x_at | MYO1C | myosin IC | down | 1.5812 | 0.0158 |  |
| 242792_at | NFIB | nuclear factor I/B | down | 1.7215 | 0.0039 |  |
| 227400_at | NFIX | Nuclear factor 1/X | down | 1.6275 | 0.0373 |  |
| 209959_at | NR4A3 | nuclear receptor subfamily 4, group A, member 3 | down | 2.0645 | 0.0435 |  |
| 223899_at | PBRM1 | polybromo 1 | down | 1.9186 | 0.0027 |  |
| 225048_at | PHF10 | PHD finger protein 10 | down | 1.5209 | 0.0041 |  |
| 209034_at | PNRC1 | Proline-rich protein 2 | down | 1.5210 | 0.0202 |  |
| 1565358_at | RARA | retinoic acid receptor alpha | down | 2.4460 | 0.0040 |  |
| 227223_at | RBM39 | RNA binding motif protein 39 | down | 1.8061 | 0.0157 | PLS |
| 232135_at | SAP30L | Sin3A-associated protein p30-like protein | down | 1.6747 | 0.0011 |  |
| 224754_at | SP1 | Sp1 transcription factor | down | 1.5074 | 0.0408 |  |
| 201996_s_at | SPEN | spen homolog, transcriptional regulator (Drosophila) | down | 1.8263 | 0.0246 | PLS |
| 235925_at | TCF12 | transcription factor 12 | down | 1.5806 | 0.0125 |  |
| 203753_at | TCF4 | transcription factor 4 | down | 1.8111 | 0.0216 |  |
| 215686_x_at | TFAP2B | transcription factor AP-2 beta | down | 1.6476 | 0.0187 |  |
| 223392_s_at | TSHZ3 | teashirt zinc finger homeobox 3 | down | 1.5020 | 0.0043 |  |
| 202173_s_at | VEZF1 | vascular endothelial zinc finger 1 | down | 1.7789 | 0.0383 |  |
| 243561_at | YAF2 | YY1 associated factor 2 | down | 1.6452 | 0.0177 |  |
| 226554_at | ZBTB7A | zinc finger and BTB domain containing 7A | down | 1.7684 | 0.0045 |  |
| 242738_s_at | ZFHX3 | zinc finger homeobox 3 | down | 1.5119 | 0.0022 |  |
| 235698_at | ZFP90 | zinc finger protein 90 | down | 1.5966 | 0.0177 | PLS |
| 219571_s_at | ZNF12 | Zinc finger protein 12 | down | 1.8199 | 0.0125 |  |
| 214741_at | ZNF131 | zinc finger protein 131 | down | 1.6680 | 0.0230 |  |
| 233399_x_at | ZNF252 | zinc finger protein 252 | down | 1.5238 | 0.0291 |  |
| 205917_at | ZNF264 | zinc finger protein 264 | down | 1.5019 | 0.0110 |  |
| 222619_at | ZNF281 | zinc finger protein 281 | down | 1.7502 | 0.0179 | PLS |
| 212368_at | ZNF292 | zinc finger protein 292 | down | 1.9234 | 0.0092 |  |
| 219228_at | ZNF331 | zinc finger protein 331 | down | 1.8069 | 0.0134 | PLS |
| 231369_at | ZNF333 | zinc finger protein 333 | down | 1.6669 | 0.0069 | PLS |
| 220117_at | ZNF385D | zinc finger protein 385D | down | 1.8616 | 0.0021 |  |
| 239619_at | ZNF395 | zinc finger protein 395 | down | 1.8109 | 0.0235 | PLS |
| 235604_x_at | ZNF493 | zinc finger protein 493 | down | 1.5496 | 0.0006 |  |
| 211721_s_at | ZNF551 | zinc finger protein 551 | down | 1.5411 | 0.0036 |  |
| 227952_at | ZNF595 | zinc finger protein 595 | down | 1.9821 | 0.0185 |  |
| 238444_at | ZNF618 | zinc finger protein 618 | down | 1.6701 | 0.0224 |  |
| 205594_at | ZNF652 | zinc finger protein 652 | down | 1.5355 | 0.0474 |  |
| 207120_at | ZNF667 | zinc finger protein 667 | down | 1.6371 | 0.0174 | PLS |
| 1553704_x_at | ZNF791 | zinc finger protein 791 | down | 1.7715 | 0.0000 |  |
| 60794_f_at | ZNF814 | zinc finger protein 814 | down | 1.9761 | 0.0162 | PLS |
| 206059_at | ZNF91 | zinc finger protein 91 | down | 1.6300 | 0.0257 |  |
| 208119_s_at | ZNF93 | zinc finger protein 93 | down | 1.6001 | 0.0055 |  |
| 217593_at | ZSCAN18 | zinc finger and SCAN domain containing 18 | down | 1.7635 | 0.0181 | PLS |
| 222544_s_at | WHSC1L1 | Wolf-Hirschhorn syndrome candidate 1-like 1 | down | 1.6823 | 0.0348 |  |
| **Unknown** | | | | | | |
| 220859_at | ADAM32 | ADAM metallopeptidase domain 32 | down | 1.7275 | 0.0013 | PLS |
| 238151_at | AFG3L1 | AFG3 ATPase family gene 3-like 2 (yeast) | down | 1.5199 | 0.0104 |  |
| 1556211_a_at | AIG1 | androgen-induced 1 | down | 1.5781 | 0.0238 |  |
| 227260_at | ANKRD10 | ankyrin repeat domain 10 | down | 1.8632 | 0.0436 |  |
| 216550_x_at | ANKRD12 | ankyrin repeat domain 12 | down | 1.6126 | 0.0213 | PLS |
| 238851_at | ANKRD13A | ankyrin repeat domain 13A | down | 1.8774 | 0.0456 |  |
| 1567361_at | BDNFOS | BDNF antisense RNA (non-protein coding) | down | 1.5257 | 0.0067 |  |
| 219670_at | BEND5 | BEN domain containing 5 | down | 2.1152 | 0.0060 | PLS |
| 229851_s_at | C11orf54 | chromosome 11 open reading frame 54 | down | 1.5819 | 0.0380 |  |
| 233106_at | C14orf82 | chromosome 14 open reading frame 82 | down | 1.8179 | 0.0146 |  |
| 227963_at | C17orf76 | chromosome 17 open reading frame 76 | down | 1.7148 | 0.0003 | PLS |
| 222266_at | C19orf2 | chromosome 19 open reading frame 2 | down | 1.5562 | 0.0291 |  |
| 221222_s_at | C1orf56 | chromosome 1 open reading frame 56 | down | 1.5209 | 0.0478 |  |
| 225825_at | C20orf194 | chromosome 20 open reading frame 194 | down | 1.6416 | 0.0179 |  |
| 228449_at | C22orf27 | chromosome 22 open reading frame 27 | down | 1.6346 | 0.0030 |  |
| 237585_at | C4orf47 | chromosome 4 open reading frame 47 | down | 1.7372 | 0.0146 | PLS |
| 230424_at | C5orf13 | chromosome 5 open reading frame 13 | down | 2.2090 | 0.0289 |  |
| 219054_at | C5orf23 | chromosome 5 open reading frame 23 | down | 1.7342 | 0.0139 | PLS |
| 238635_at | C5orf28 | chromosome 5 open reading frame 28 | down | 1.7006 | 0.0310 |  |
| 230251_at | C6orf176 | chromosome 6 open reading frame 176 | down | 2.0479 | 0.0131 |  |
| 1553840_a_at | CCDC149 | coiled-coil domain containing 149 | up | 1.6980 | 0.0415 |  |
| 242949_x_at | CCDC157 | coiled-coil domain containing 157 | down | 1.6681 | 0.0240 | PLS |
| 1552750_at | CIB3 | calcium and integrin binding family member 3 | up | 1.7387 | 0.0293 |  |
| 219468_s_at | CUEDC1 | CUE domain containing 1 | down | 1.6996 | 0.0201 |  |
| 228739_at | CYS1 | cystin 1 | down | 1.5376 | 0.0302 |  |
| 232356_at | EFR3A | EFR3 homolog A | down | 1.5226 | 0.0294 |  |
| 226876_at | FAM101B | family with sequence similarity 101, member B | down | 1.9261 | 0.0066 | PLS |
| 213861_s_at | FAM119B | family with sequence similarity 119, member B | down | 1.7459 | 0.0086 | PLS |
| 220720_x_at | FAM128B | family with sequence similarity 128, member B | down | 1.5103 | 0.0448 |  |
| 235105_at | FAM184A | family with sequence similarity 184, member B | down | 1.6732 | 0.0028 | PLS |
| 206848_at | FAM36A | family with sequence similarity 36, member A | down | 1.5532 | 0.0030 |  |
| 229737_at | FAM46A | Family with sequence similarity 46, member A | down | 2.2040 | 0.0008 | PLS |
| 230817_at | FAM84B | family with sequence similarity 84, member B | down | 1.6339 | 0.0065 | PLS |
| 239487_at | FAM98A | family with sequence similarity 98, member A | down | 1.9444 | 0.0315 |  |
| 225704_at | FBRSL1 | fibrosin-like 1 | down | 1.5361 | 0.0002 |  |
| 240042_at | FIBCD1 | fibrinogen C domain containing 1 | up | 1.6156 | 0.0439 |  |
| 211454_x_at | FKSG49 | FKSG49 | down | 1.5348 | 0.0070 |  |
| 232498_at | HEATR7A | HEAT repeat containing 7A | down | 1.7564 | 0.0153 |  |
| 1553793_a_at | KIAA1109 | KIAA1109 | up | 1.6426 | 0.0493 |  |
| 223161_at | KIAA1147 | KIAA1147 | up | 1.7438 | 0.0402 |  |
| 231807_at | KIAA1217 | KIAA1217 | up | 1.5710 | 0.0209 |  |
| 1557961_s_at | LOC100127983 | hypothetical protein LOC100127983 | down | 2.0054 | 0.0107 | PLS |
| 237772_at | LOC100129286 | tetratricopeptide repeat domain 7A | down | 1.6299 | 0.0092 |  |
| 238771_at | LOC100129899 | hypothetical LOC100129899 | down | 1.5850 | 0.0069 |  |
| 214182_at | LOC100132430 | hypothetical LOC100132430 | down | 3.2150 | 0.0265 |  |
| 1556750_at | LOC153577 | hypothetical LOC153577 | down | 1.6821 | 0.0476 |  |
| 238536_at | LOC286272 | hypothetical protein LOC286272 | down | 1.5230 | 0.0425 |  |
| 241822_at | LOC388553 | hypothetical LOC388553 | down | 1.6230 | 0.0034 |  |
| 232113_at | LOC399959 | hypothetical LOC399959 | down | 1.7053 | 0.0079 |  |
| 242546_at | LOC642477 | hypothetical LOC642477 | up | 1.5804 | 0.0262 |  |
| 1559957_a_at | LOC642852 | hypothetical LOC642852 | down | 1.7901 | 0.0157 | PLS |
| 1561180_at | LRP11 | low density lipoprotein receptor-related protein 11 | down | 1.5233 | 0.0092 |  |
| 230793_at | LRRC16A | leucine rich repeat containing 16A | down | 2.0135 | 0.0037 | PLS |
| 240814_at | MGC39584 | hypothetical gene supported by BC029568 | down | 1.5200 | 0.0302 |  |
| 238502_at | MORF4L2 | mortality factor 4 like 2 | down | 1.5817 | 0.0446 |  |
| 235547_at | N4BP2L2 | NEDD4 binding protein 2-like 2 | down | 2.0103 | 0.0025 | PLS |
| 224770_s_at | NAV1 | neuron navigator 1 | down | 1.6452 | 0.0127 | PLS |
| 213788_s_at | NCRNA00094 | non-protein coding RNA 94 | down | 1.8698 | 0.0332 |  |
| 226140_s_at | OTUD1 | OTU domain containing 1 | down | 1.8740 | 0.0031 | PLS |
| 229413_s_at | PCGF3 | polycomb group ring finger 3 | down | 1.5310 | 0.0041 |  |
| 227858_at | PCNXL3 | pecanex-like 3 | up | 1.5551 | 0.0066 |  |
| 237477_at | PDCL2 | phosducin-like 2 | down | 1.8185 | 0.0039 |  |
| 205251_at | PER2 | period circadian protein 2 | down | 1.6450 | 0.0249 |  |
| 220014_at | PRR16 | proline rich 16 | down | 1.6181 | 0.0349 |  |
| 218967_s_at | PTER | phosphotriesterase related | down | 1.5389 | 0.0020 | PLS |
| 243041_s_at | RBMS3 | RNA binding motif, single stranded interacting protein 3 | down | 2.0314 | 0.0192 | PLS |
| 231716_at | RC3H2 | ring finger and CCCH-type zinc finger domains 2 | down | 1.7997 | 0.0459 |  |
| 237062_at | RNF10 | ring finger protein 10 | down | 1.7742 | 0.0128 | PLS |
| 222791_at | RSBN1 | round spermatid basic protein 1 | down | 1.6491 | 0.0398 |  |
| 239022_at | SDHAP1 | succinate dehydrogenase complex, subunit A, flavoprotein pseudogene 1 | down | 1.5543 | 0.0032 |  |
| 226763_at | SESTD1 | SEC14 and spectrin domains 1 | down | 1.5377 | 0.0007 |  |
| 1559469_s_at | SIPA1L2 | signal-induced proliferation-associated 1 like 2 | up | 1.6649 | 0.0386 |  |
| 1558832_at | SLC2A1-AS1 | SLC2A1 antisense RNA 1 (non-protein coding) | down | 1.5321 | 0.0277 |  |
| 1561886_a_at | SLC39A14 | solute carrier family 39 (zinc transporter), member 14 | down | 2.0884 | 0.0034 |  |
| 1553020_at | SMCR5 | Smith-Magenis syndrome chromosome region, candidate 5 (non-protein coding) | down | 2.0143 | 0.0034 | PLS |
| 226913_s_at | SOX8 | SRY (sex determining region Y)-box 8 | down | 1.5724 | 0.0086 | PLS |
| 214965_at | SPATA2L | spermatogenesis associated 2-like | down | 1.5288 | 0.0092 |  |
| 222532_at | SRPRB | signal recognition particle receptor, B subunit | up | 1.6016 | 0.0223 | PLS |
| 233383_at | TBC1D5 | TBC1 domain family, member 5 | down | 1.9440 | 0.0025 | PLS |
| 206555_s_at | THUMPD1 | THUMP domain containing 1 | down | 1.6568 | 0.0390 |  |
| 229126_at | TMEM19 | transmembrane protein 19 | up | 1.5049 | 0.0061 | PLS |
| 224159_x_at | TRIM4 | tripartite motif-containing 4 | down | 1.5229 | 0.0157 |  |
| 235476_at | TRIM59 | tripartite motif-containing 59 | down | 1.8288 | 0.0450 |  |
| 208663_s_at | TTC3 | tetratricopeptide repeat domain 3 | down | 1.6161 | 0.0029 |  |
| 221064_s_at | UNKL | unkempt homolog (Drosophila)-like | down | 1.5419 | 0.0311 |  |
| 1552396_at | WFDC6 | WAP four-disulfide core domain 6 | down | 1.8327 | 0.0025 |  |
| 221939_at | YIPF2 | Yip1 domain family, member 2 | down | 1.6006 | 0.0120 |  |
| 224503_s_at | ZCCHC2 | zinc finger, CCHC domain containing 2 | down | 1.5969 | 0.0062 |  |
| 1553736_at | ZFC3H1 | zinc finger, C3H1-type containing | down | 1.5939 | 0.0072 |  |
| 204893_s_at | ZFYVE9 | zinc finger, FYVE domain containing 9 | down | 1.5114 | 0.0192 |  |
| 236310_at | ZNF37B | zinc finger protein 37B | down | 1.6349 | 0.0073 | PLS |
| 241906_at | ZNF708 | zinc finger protein 708 | down | 1.5066 | 0.0315 |  |
| 1568817_at |  | unknown | down | 2.0670 | 0.0029 | PLS |
| 217435_x_at |  | unknown | down | 1.6604 | 0.0056 | PLS |
| 222288_at |  | unknown | down | 1.9402 | 0.0091 | PLS |
| 235251_at |  | unknown | up | 1.8353 | 0.0009 | PLS |
| 237263_at |  | unknown | up | 1.9285 | 0.0027 | PLS |
| 238847_at |  | unknown | down | 1.7592 | 0.0057 | PLS |
| 1555373_at |  | unknown | down | 1.5825 | 0.0026 |  |
| 1560962_at |  | unknown | up | 1.8319 | 0.0014 |  |
| 1563107_at |  | unknown | down | 1.6704 | 0.0021 |  |
| 214808_at |  | unknown | down | 1.5291 | 0.0001 |  |
| 215287_at |  | unknown | down | 1.5147 | 0.0177 |  |
| 220494_s_at |  | unknown | down | 1.9877 | 0.0340 |  |
| 222145_at |  | unknown | down | 1.6503 | 0.0167 |  |
| 228156_at |  | unknown | down | 1.8093 | 0.0449 |  |
| 228812_at |  | unknown | down | 1.5676 | 0.0167 |  |
| 231489_x_at |  | unknown | down | 1.5043 | 0.0014 |  |
| 232804_at |  | unknown | down | 2.0508 | 0.0012 |  |
| 232822_x_at |  | unknown | down | 1.5188 | 0.0067 |  |
| 233518_at |  | unknown | down | 1.5921 | 0.0464 |  |
| 233775_x_at |  | unknown | down | 1.5128 | 0.0426 |  |
| 234578_at |  | unknown | down | 1.5208 | 0.0138 |  |
| 234675_x_at |  | unknown | down | 1.6426 | 0.0220 |  |
| 235242_at |  | unknown | down | 1.6990 | 0.0000 |  |
| 239154_at |  | unknown | down | 1.5262 | 0.0280 |  |
| 239973_at |  | unknown | down | 1.5004 | 0.0181 |  |
| 241227_at |  | unknown | down | 1.9259 | 0.0032 |  |
| 241625_at |  | unknown | down | 1.7491 | 0.0029 |  |
| 241632_x_at |  | unknown | down | 1.6021 | 0.0036 |  |
| 242358_at |  | unknown | down | 1.6524 | 0.0221 |  |
| 242471_at |  | unknown | down | 1.5932 | 0.0092 |  |
| 242862_x_at |  | unknown | down | 1.5599 | 0.0059 |  |
| 244018_at |  | unknown | down | 1.5059 | 0.0106 |  |
| 244433_at |  | unknown | down | 1.5833 | 0.0166 |  |
