# Supplementary material for: Gene expression signatures in motor neurone disease fibroblasts reveal dysregulation of metabolism, hypoxia-response and RNA processing functions
Source: Neuropathol Appl Neurobiol. 2015 Jan 29;41(2):201–26. doi: 10.1111/nan.12147 (PMC4329387; doi:10.1111/nan.12147)
Supplement: Table S4 — List of differentially expressed gene in PLS fibroblasts compared to controls categorized according to biological function [file nan0041-0201-sd4.docx]

**Supplementary Table 4:** List of differentially expressed gene in PLS fibroblasts compared to controls categorised according to biological function.

| **Probe Set ID** | **Gene Symbol** | **Gene Name** | **Regulation** | **Fold Change** | **p-value** | **Altered in ALS?** |
| --- | --- | --- | --- | --- | --- | --- |
| **Apoptosis and Cell Survival** | | | | | | |
| 203685_at | BCL2 | B-cell CLL/lymphoma 2 | up | 1.5130 | 0.0062 |  |
| 210334_x_at | BIRC5 | baculoviral IAP repeat-containing 5 | up | 1.7166 | 0.0203 | ALS |
| 217999_s_at | PHLDA1 | pleckstrin homology-like domain, family A, member 1 | up | 1.6643 | 0.0136 | ALS |
| **Cell Adhesion** | | | | | | |
| 228498_at | B4GALT1 | UDP-Gal:betaGlcNAc beta 1,4- galactosyltransferase, polypeptide 1 | down | 1.6005 | 0.0019 |  |
| 211964_at | COL4A2 | collagen, type IV, alpha 2 | down | 1.7797 | 0.0493 |  |
| 229779_at | COL4A4 | collagen, type IV, alpha 4 | down | 2.1751 | 0.0280 |  |
| 203325_s_at | COL5A1 | collagen, type V, alpha 1 | down | 2.2116 | 0.0215 |  |
| 221730_at | COL5A2 | collagen, type V, alpha 2 | down | 1.7062 | 0.0166 |  |
| 201059_at | CTTN | cortactin | up | 2.0987 | 0.0235 |  |
| 212253_x_at | DST | dystonin | down | 1.5164 | 0.0055 |  |
| 201843_s_at | EFEMP1 | EGF-containing fibulin-like extracellular matrix protein 1 | down | 5.7187 | 0.0034 |  |
| 229247_at | FBLN7 | fibulin 7 | down | 1.5968 | 0.0051 |  |
| 223730_at | GPC6 | glypican 6 | down | 1.8761 | 0.0349 |  |
| 227314_at | ITGA2 | integrin, alpha 2 (CD49B, alpha 2 subunit of VLA-2 receptor) | up | 1.7557 | 0.0228 |  |
| 219213_at | JAM2 | junctional adhesion molecule 2 | down | 1.6089 | 0.0015 | ALS |
| 208933_s_at | LGALS8 | lectin, galactoside-binding, soluble, 8 | down | 1.5029 | 0.0381 | ALS |
| 205680_at | MMP10 | matrix metallopeptidase 10 (stromelysin 2) | up | 3.8622 | 0.0218 |  |
| 204580_at | MMP12 | matrix metallopeptidase 12 (macrophage elastase) | up | 1.5133 | 0.0489 | ALS |
| 205828_at | MMP3 | matrix metallopeptidase 3 (stromelysin 1, progelatinase) | up | 6.1955 | 0.0110 | ALS |
| 211432_s_at | TYRO3 | TYRO3 protein tyrosine kinase | up | 1.7428 | 0.0117 |  |
| **Cell Cycle** | | | | | | |
| 226611_s_at | CENPV | centromere protein V | down | 1.6419 | 0.0062 | ALS |
| 205022_s_at | FOXN3 | forkhead box N3 | down | 1.6993 | 0.0139 | ALS |
| 212530_at | NEK7 | NIMA (never in mitosis gene a)-related kinase 7 | down | 1.6631 | 0.0445 |  |
| 210543_s_at | PRKDC | protein kinase, DNA-activated, catalytic polypeptide | up | 1.5761 | 0.0449 |  |
| 212413_at | SEPT6 | septin 6 | down | 1.7116 | 0.0120 | ALS |
| 227862_at | TRNP1 | TMF1-regulated nuclear protein 1 | up | 1.6085 | 0.0049 |  |
| **Cytoskeleton** | | | | | | |
| 217818_s_at | ARPC4 | actin related protein 2/3 complex, subunit 4, 20kDa | up | 1.7003 | 0.0440 |  |
| 228297_at | CNN3 | calponin 3, acidic | down | 4.4207 | 0.0229 |  |
| 205493_s_at | DPYSL4 | dihydropyrimidinase-like 4 | down | 2.3422 | 0.0103 | ALS |
| 215017_s_at | FNBP1L | formin binding protein 1-like | down | 1.6635 | 0.0215 |  |
| 224469_s_at | INF2 | inverted formin, FH2 and WH2 domain containing | up | 1.5306 | 0.0286 |  |
| 213201_s_at | TNNT1 | troponin T type 1 (skeletal, slow) | down | 2.0938 | 0.0062 | ALS |
| **Immune Response** | | | | | | |
| 223710_at | CCL26 | chemokine (C-C motif) ligand 26 | up | 1.6999 | 0.0021 | ALS |
| 215388_s_at | CFH /// CFHR1 | complement factor H /// complement factor H-related 1 | up | 1.7255 | 0.0255 |  |
| 214012_at | ERAP1 | endoplasmic reticulum aminopeptidase 1 | down | 2.2773 | 0.0093 |  |
| 214022_s_at | IFITM1 | interferon induced transmembrane protein 1 (9-27) | down | 1.8997 | 0.0143 | ALS |
| 211632_at | IGHD | immunoglobulin heavy constant delta | down | 1.5445 | 0.0017 | ALS |
| 205798_at | IL7R | interleukin 7 receptor | down | 2.4736 | 0.0452 |  |
| 205904_at | MICA | MHC class I polypeptide-related sequence A | up | 2.1357 | 0.0042 | ALS |
| **Ion Transport** | | | | | | |
| 207103_at | KCND2 | potassium voltage-gated channel, Shal-related subfamily, member 2 | down | 1.6926 | 0.0149 | ALS |
| 222379_at | KCNE4 | potassium voltage-gated channel, Isk-related family, member 4 | down | 1.8855 | 0.0434 |  |
| 235857_at | KCTD11 | potassium channel tetramerisation domain containing 11 | down | 1.7882 | 0.0215 | ALS |
| 223176_at | KCTD20 | potassium channel tetramerisation domain containing 20 | down | 1.8792 | 0.0086 | ALS |
| **Metabolism** | | | | | | |
| 202587_s_at | AK1 | adenylate kinase 1 | up | 1.6176 | 0.0001 | ALS |
| 230630_at | AK4 | adenylate kinase 4 | down | 1.6673 | 0.0155 | ALS |
| 203946_s_at | ARG2 | arginase, type II | down | 1.6331 | 0.0359 |  |
| 206756_at | CHST7 | carbohydrate (N-acetylglucosamine 6-O) sulfotransferase 7 | up | 1.5012 | 0.0263 |  |
| 204646_at | DPYD | dihydropyrimidine dehydrogenase | down | 1.6059 | 0.0119 |  |
| 213787_s_at | EBP | emopamil binding protein (sterol isomerase) | up | 1.7398 | 0.0053 | ALS |
| 202218_s_at | FADS2 | fatty acid desaturase 2 | down | 1.8013 | 0.0003 | ALS |
| 227377_at | IGF2BP1 | insulin-like growth factor 2 mRNA binding protein 1 | up | 2.4319 | 0.0034 | ALS |
| 202718_at | IGFBP2 | insulin-like growth factor binding protein 2, 36kDa | up | 2.3782 | 0.0272 |  |
| 209566_at | INSIG2 | insulin induced gene 2 | down | 1.5578 | 0.0158 |  |
| 227432_s_at | IR | insulin receptor | down | 2.4138 | 0.0120 | ALS |
| 209184_s_at | IRS2 | insulin receptor substrate 2 | down | 1.7038 | 0.0288 |  |
| 226726_at | MBOAT2 | membrane bound O-acyltransferase domain containing 2 | down | 1.6300 | 0.0397 |  |
| 213607_x_at | NADK | NAD kinase | up | 1.6066 | 0.0283 |  |
| 201577_at | NME1 | non-metastatic cells 1, protein (NM23A) expressed in | up | 1.5303 | 0.0018 | ALS |
| 204491_at | PDE4D | phosphodiesterase 4D, cAMP-specific | down | 2.2035 | 0.0242 |  |
| 230109_at | PDE7B | phosphodiesterase 7B | down | 1.5540 | 0.0279 | ALS |
| 229553_at | PGM2L1 | phosphoglucomutase 2-like 1 | down | 1.6498 | 0.0101 | ALS |
| 1554997_a_at | PTGS2 | prostaglandin-endoperoxide synthase 2 (prostaglandin G/H synthase and cyclooxygenase) | down | 3.3941 | 0.0108 |  |
| 220232_at | SCD5 | stearoyl-CoA desaturase 5 | down | 1.6296 | 0.0071 | ALS |
| 230748_at | SLC16A6 | solute carrier family 16, member 6 (monocarboxylic acid transporter 7) | up | 1.7984 | 0.0310 |  |
| 201918_at | SLC25A36 | solute carrier family 25, member 36 | down | 1.5935 | 0.0015 | ALS |
| 225779_at | SLC27A4/FATP4 | solute carrier family 27 (fatty acid transporter), member 4 | up | 1.5701 | 0.0064 | ALS |
| 202499_s_at | SLC2A3 | solute carrier family 2 (facilitated glucose transporter), member 3 | down | 1.8762 | 0.0027 | ALS |
| 212353_at | SULF1 | sulfatase 1 | down | 3.2156 | 0.0098 |  |
| **Miscellaneous** | | | | | | |
| 242693_at | CDK13 | cyclin dependent kinase 13 | down | 1.6592 | 0.0043 | ALS |
| 229171_at | CENPBD1 | CENPB DNA-binding domains containing 1 | down | 1.5856 | 0.0222 | ALS |
| 1554241_at | COCH | coagulation factor C homolog, cochlin (Limulus polyphemus) | down | 1.8930 | 0.0316 | ALS |
| 215252_at | DNAJC7 | DnaJ (Hsp40) homolog, subfamily C, member 7 | down | 1.6569 | 0.0116 | ALS |
| 214718_at | GATAD1 | GATA zinc finger domain containing 1 | down | 1.5232 | 0.0133 | ALS |
| 235371_at | GLT8D4 | glycosyltransferase 8 domain containing 4 | down | 1.8836 | 0.0093 | ALS |
| 229400_at | HOXD10 | homeobox D10 | down | 1.5147 | 0.0137 | ALS |
| 207180_s_at | HTATIP2 | HIV-1 Tat interactive protein 2, 30kDa | up | 1.8362 | 0.0234 |  |
| 238119_at | MFF | mitochondrial fission factor | down | 1.8435 | 0.0100 | ALS |
| 211496_s_at | PDC | phosducin | down | 1.9999 | 0.0054 | ALS |
| 218901_at | PLSCR4 | phospholipid scramblase 4 | down | 1.9371 | 0.0015 | ALS |
| 222653_at | PNPO | pyridoxamine 5'-phosphate oxidase | up | 1.5287 | 0.0013 | ALS |
| 209723_at | SERPINB9 | serpin peptidase inhibitor, clade B (ovalbumin), member 9 | down | 1.6762 | 0.0238 | ALS |
| 1559515_at | XPA | xeroderma pigmentosum, complementation group A | down | 1.5647 | 0.0021 | ALS |
| **Protein Catabolism** | | | | | | |
| 236235_at | ITCH | Itchy E3 ubiquitin protein ligase homolog (mouse) | down | 2.0052 | 0.0189 | ALS |
| 233819_s_at | LTN1 | listerin E3 ubiquitin protein ligase 1 | down | 1.6938 | 0.0187 | ALS |
| 229441_at | PRSS23 | Protease, serine, 23 | down | 2.0300 | 0.0276 |  |
| 201400_at | PSMB3 | proteasome (prosome, macropain) subunit, beta type, 3 | up | 1.5050 | 0.0069 |  |
| 232033_at | USP37 | ubiquitin specific peptidase 37 | down | 1.5005 | 0.0146 |  |
| 210561_s_at | WSB1 | WD repeat and SOCS box-containing 1 | down | 1.5152 | 0.0118 | ALS |
| **Protein Transport** | | | | | | |
| 232627_at | HGS | hepatocyte growth factor regulated tyrosine kinase substrate | down | 1.7963 | 0.0120 |  |
| 202975_s_at | RHOBTB3 | Rho-related BTB domain containing 3 | down | 1.7878 | 0.0053 | ALS |
| 217960_s_at | TOMM22 | translocase of outer mitochondrial membrane 22 homolog (yeast) | up | 1.5834 | 0.0089 | ALS |
| 202893_at | UNC13B | unc-13 homolog B (C. elegans) | up | 1.6201 | 0.0076 |  |
| **Response to Stress** | | | | | | |
| 231016_s_at | ARNT/HIF1B | Aryl hydrocarbon receptor nuclear translocator | down | 2.1533 | 0.0174 | ALS |
| 202986_at | ARNT2 | aryl-hydrocarbon receptor nuclear translocator 2 | down | 1.6444 | 0.0078 | ALS |
| **RNA Processing** | | | | | | |
| 203865_s_at | ADARB1 | adenosine deaminase, RNA-specific, B1 | down | 2.3231 | 0.0213 |  |
| 229841_at | AGO2/EIF2C2 | eukaryotic translation initiation factor 2C 2 | down | 2.2222 | 0.0111 | ALS |
| 212563_at | BOP1 | block of proliferation 1 | up | 1.5343 | 0.0174 |  |
| 201514_s_at | G3BP1 | GTPase activating protein (SH3 domain) binding protein 1 | up | 1.5318 | 0.0235 | ALS |
| 222040_at | HNRNPA1 | heterogeneous nuclear ribonucleoprotein A1 | up | 1.5575 | 0.0123 |  |
| 226134_s_at | MSI2 | musashi homolog 2 (Drosophila) | up | 1.5008 | 0.0455 |  |
| 212177_at | SFRS18 | splicing factor, arginine/serine-rich 18 | down | 1.5357 | 0.0059 | ALS |
| 228483_s_at | TAF9B | TAF9B RNA polymerase II, TATA box binding protein (TBP)-associated factor | down | 2.1397 | 0.0060 | ALS |
| **Signalling** | | | | | | |
| 229307_at | ANKRD28 | ankyrin repeat domain 28 | down | 2.4396 | 0.0251 | ALS |
| 212135_s_at | ATP2B4 | ATPase, Ca++ transporting, plasma membrane 4 | up | 1.5799 | 0.0362 |  |
| 218309_at | CAMK2N1 | calcium/calmodulin-dependent protein kinase II inhibitor 1 | up | 1.5184 | 0.0423 |  |
| 1554411_at | CTNNB1 | catenin (cadherin-associated protein), beta 1, 88kDa | up | 1.9113 | 0.0339 |  |
| 214240_at | GAL | galanin prepropeptide | up | 2.3846 | 0.0016 | ALS |
| 235851_s_at | GNAS | guanine nucleotide binding protein (G protein), alpha | down | 1.9219 | 0.0093 | ALS |
| 65718_at | GPR124 | G protein-coupled receptor 124 | down | 1.5861 | 0.0046 | ALS |
| 213094_at | GPR126 | G protein-coupled receptor 126 | down | 1.9525 | 0.0385 |  |
| 201189_s_at | ITPR3 | inositol 1,4,5-triphosphate receptor, type 3 | up | 1.6600 | 0.0201 |  |
| 1566267_at | LTBP1 | latent transforming growth factor beta binding protein 1 | down | 1.6132 | 0.0411 |  |
| 235421_at | MAP3K8 | Mitogen-activated protein kinase kinase kinase 8 | down | 1.6850 | 0.0291 |  |
| 202787_s_at | MAPKAPK3 | mitogen-activated protein kinase-activated protein kinase 3 | up | 1.5276 | 0.0299 |  |
| 1558220_at | MUC20 | Mucin 20, cell surface associated | down | 1.5076 | 0.0080 | ALS |
| 219789_at | NPR3 | natriuretic peptide receptor C/guanylate cyclase C (atrionatriuretic peptide receptor C) | down | 2.3036 | 0.0015 | ALS |
| 204589_at | NUAK1 | NUAK family, SNF1-like kinase, 1 | down | 2.1141 | 0.0363 |  |
| 226864_at | PKIA | protein kinase (cAMP-dependent, catalytic) inhibitor alpha | up | 1.5347 | 0.0016 |  |
| 228104_at | PLXNA4 | plexin A4 | up | 1.6842 | 0.0340 | ALS |
| 209678_s_at | PRKCI | protein kinase C, iota | down | 1.6699 | 0.0415 |  |
| 209815_at | PTCH1 | patched homolog 1 (Drosophila) | down | 1.8284 | 0.0006 | ALS |
| 227340_s_at | RGMB | RGM domain family, member B | up | 1.5781 | 0.0445 |  |
| 201614_s_at | RUVBL1 | RuvB-like 1 (E. coli) | up | 1.5371 | 0.0211 |  |
| 226492_at | SEMA6D | sema domain, transmembrane domain (TM), and cytoplasmic domain, (semaphorin) 6D | up | 1.5335 | 0.0256 |  |
| 235518_at | SLC8A1/NCX1 | solute carrier family 8 (sodium/calcium exchanger), member 1 | down | 1.8253 | 0.0034 |  |
| 221016_s_at | TCF7L1 | transcription factor 7-like 1 (T-cell specific, HMG-box) | down | 2.0210 | 0.0010 | ALS |
| 212762_s_at | TCF7L2 | transcription factor 7-like 2 (T-cell specific, HMG-box) | down | 1.8661 | 0.0017 | ALS |
| 222736_s_at | TMEM38B | transmembrane protein 38B | up | 1.6385 | 0.0038 | ALS |
| **Transcription** | | | | | | |
| 219437_s_at | ANKRD11 | ankyrin repeat domain 11 | down | 1.8220 | 0.0138 | ALS |
| 231090_s_at | ARID2 | AT rich interactive domain 2 (ARID, RFX-like) | down | 1.8504 | 0.0229 | ALS |
| 222891_s_at | BCL11A | B-cell CLL/lymphoma 11A (zinc finger protein) | down | 1.6181 | 0.0109 |  |
| 204908_s_at | BCL3 | B-cell CLL/lymphoma 3 | down | 1.5757 | 0.0078 | ALS |
| 239364_at | Etv6 | ets variant 6 | down | 2.1168 | 0.0080 | ALS |
| 220102_at | FOXL2 | forkhead box L2 | up | 1.7454 | 0.0403 |  |
| 224837_at | FOXP1 | forkhead box P1 | down | 1.6292 | 0.0078 | ALS |
| 208546_x_at | HIST1H2BH | histone cluster 1, H2bh | up | 1.5742 | 0.0130 | ALS |
| 206289_at | HOXA4 | homeobox A4 | down | 1.6271 | 0.0207 |  |
| 213003_s_at | KIAA0146 | KIAA0146 | down | 1.9139 | 0.0231 |  |
| 203542_s_at | KLF9 | Kruppel-like factor 9 | down | 1.7611 | 0.0023 | ALS |
| 236241_at | MED31 | mediator complex subunit 31 | down | 1.5894 | 0.0006 | ALS |
| 202364_at | MXI1 | MAX interactor 1 | down | 1.6028 | 0.0097 | ALS |
| 218849_s_at | PPP1R13L | protein phosphatase 1, regulatory (inhibitor) subunit 13 like | down | 1.7563 | 0.0294 |  |
| 227223_at | RBM39 | RNA binding motif protein 39 | down | 1.6165 | 0.0157 | ALS |
| 235567_at | RORA | RAR-related orphan receptor A | down | 1.5972 | 0.0136 |  |
| 201996_s_at | SPEN | spen homolog, transcriptional regulator (Drosophila) | down | 1.8206 | 0.0246 | ALS |
| 235698_at | ZFP90 | zinc finger protein 90 homolog (mouse) | down | 1.5298 | 0.0177 | ALS |
| 222619_at | ZNF281 | zinc finger protein 281 | down | 1.6206 | 0.0179 | ALS |
| 219228_at | ZNF331 | zinc finger protein 331 | down | 1.6224 | 0.0134 | ALS |
| 231369_at | ZNF333 | Zinc finger protein 333 | down | 1.6708 | 0.0069 | ALS |
| 239619_at | ZNF395 | zinc finger protein 395 | down | 1.7438 | 0.0235 | ALS |
| 207120_at | ZNF667 | zinc finger protein 667 | down | 1.5371 | 0.0174 | ALS |
| 60794_f_at | ZNF814 | zinc finger protein 814 | down | 1.9362 | 0.0162 | ALS |
| 217593_at | ZSCAN18 | zinc finger and SCAN domain containing 18 | down | 1.6778 | 0.0181 | ALS |
| **Unknown** | | | | | | |
| 220859_at | ADAM32 | ADAM metallopeptidase domain 32 | down | 1.5234 | 0.0013 | ALS |
| 216550_x_at | ANKRD12 | ankyrin repeat domain 12 | down | 1.5792 | 0.0213 | ALS |
| 219670_at | BEND5 | BEN domain containing 5 | down | 2.4689 | 0.0060 | ALS |
| 201261_x_at | BGN | biglycan | down | 1.6466 | 0.0030 |  |
| 227963_at | C17orf76 | chromosome 17 open reading frame 76 | down | 2.1691 | 0.0003 | ALS |
| 237585_at | C4orf47 | chromosome 4 open reading frame 47 | down | 1.6521 | 0.0146 | ALS |
| 219054_at | C5orf23 | chromosome 5 open reading frame 23 | down | 1.7951 | 0.0139 | ALS |
| 242949_x_at | CCDC157 | Coiled-coil domain containing 157 | down | 1.6098 | 0.0240 | ALS |
| 226876_at | FAM101B | family with sequence similarity 101, member B | down | 1.6582 | 0.0066 | ALS |
| 213861_s_at | FAM119B | family with sequence similarity 119, member B | down | 1.6656 | 0.0086 | ALS |
| 235105_at | FAM184A | family with sequence similarity 184, member B | down | 2.2321 | 0.0028 | ALS |
| 229737_at | FAM46A | Family with sequence similarity 46, member A | down | 1.6652 | 0.0008 | ALS |
| 230817_at | FAM84B | family with sequence similarity 84, member B | down | 1.7134 | 0.0065 | ALS |
| 1557961_s_at | LOC100127983 | hypothetical protein LOC100127983 | down | 1.9170 | 0.0107 | ALS |
| 238901_at | LOC100505633 | hypothetical LOC100505633 | down | 1.7286 | 0.0203 |  |
| 243641_at | LOC100506266 | hypothetical LOC100506266 | down | 2.0684 | 0.0189 |  |
| 231233_at | LOC100506696 | hypothetical LOC100506696 | down | 1.5002 | 0.0232 |  |
| 235557_at | LOC150763 | glycerol-3-phosphate acyltransferase 2, mitochondrial | down | 2.6839 | 0.0166 |  |
| 230937_at | LOC285835 | hypothetical protein LOC285835 | up | 2.0370 | 0.0083 |  |
| 1559957_a_at | LOC642852 | hypothetical LOC642852 | down | 1.8226 | 0.0157 | ALS |
| 230793_at | LRRC16A | leucine rich repeat containing 16A | down | 1.8174 | 0.0037 | ALS |
| 226748_at | LYSMD2 | LysM, putative peptidoglycan-binding, domain containing 2 | up | 1.5425 | 0.0207 |  |
| 228327_x_at | MEIS3 | Meis homeobox 3 | down | 1.5321 | 0.0247 |  |
| 229033_s_at | MUM1 | melanoma associated antigen (mutated) 1 | down | 1.5888 | 0.0296 |  |
| 235547_at | N4BP2L2 | NEDD4 binding protein 2-like 2 | down | 1.7340 | 0.0025 | ALS |
| 224770_s_at | NAV1 | neuron navigator 1 | down | 1.5724 | 0.0127 | ALS |
| 226140_s_at | OTUD1 | OTU domain containing 1 | down | 1.7864 | 0.0031 | ALS |
| 218967_s_at | PTER | phosphotriesterase related | down | 1.9117 | 0.0020 | ALS |
| 243041_s_at | RBMS3 | RNA binding motif, single stranded interacting protein 3 | down | 2.0330 | 0.0192 | ALS |
| 237062_at | RNF10 | ring finger protein 10 | down | 1.6465 | 0.0128 | ALS |
| 228274_at | SDLS | serine dehydratase-like | up | 1.5313 | 0.0193 |  |
| 1553020_at | SMCR5 | Smith-Magenis syndrome chromosome region, candidate 5 (non-protein coding) | down | 1.6229 | 0.0034 | ALS |
| 226913_s_at | SOX8 | SRY (sex determining region Y)-box 8 | down | 1.7907 | 0.0086 | ALS |
| 222532_at | SRPRB | signal recognition particle receptor, B subunit | up | 1.5308 | 0.0223 | ALS |
| 233383_at | TBC1D5 | TBC1 domain family member 5 | down | 1.5373 | 0.0025 | ALS |
| 241359_at | TLDC2 | TLC domain containing 2 | down | 1.5186 | 0.0030 |  |
| 229126_at | TMEM19 | transmembrane protein 19 | up | 1.5045 | 0.0061 | ALS |
| 231697_s_at | TMEM49 | transmembrane protein 49 | down | 1.7049 | 0.0239 |  |
| 225412_at | TMEM87B | transmembrane protein 87B | down | 1.5102 | 0.0014 |  |
| 231393_x_at | ZBTB43 | Zinc finger and BTB domain containing 43 | down | 1.8164 | 0.0423 |  |
| 236310_at | ZNF37B | zinc finger protein 37B | down | 1.6536 | 0.0073 | ALS |
| 1556138_a_at |  | unknown | up | 1.5072 | 0.0115 |  |
| 1565856_at |  | unknown | up | 1.5108 | 0.0071 |  |
| 1568817_at |  | unknown | down | 1.5807 | 0.0029 | ALS |
| 215206_at |  | unknown | down | 1.7271 | 0.0330 |  |
| 217435_x_at |  | unknown | down | 1.6670 | 0.0056 | ALS |
| 222288_at |  | unknown | down | 1.8098 | 0.0091 | ALS |
| 231314_at |  | unknown | up | 1.7680 | 0.0259 |  |
| 232501_at |  | unknown | up | 2.2531 | 0.0147 |  |
| 235251_at |  | unknown | up | 3.0825 | 0.0009 | ALS |
| 237263_at |  | unknown | up | 1.6955 | 0.0027 | ALS |
| 238032_at |  | unknown | down | 1.5257 | 0.0217 |  |
| 238651_at |  | unknown | down | 1.5594 | 0.0076 |  |
| 238847_at |  | unknown | down | 1.7086 | 0.0057 | ALS |
| 241864_x_at |  | unknown | down | 1.5468 | 0.0348 |  |
| 244677_at |  | unknown | down | 1.5221 | 0.0122 |  |
