# Supplementary material for: Gene expression signatures in motor neurone disease fibroblasts reveal dysregulation of metabolism, hypoxia-response and RNA processing functions
Source: Neuropathol Appl Neurobiol. 2015 Jan 29;41(2):201–26. doi: 10.1111/nan.12147 (PMC4329387; doi:10.1111/nan.12147)
Supplement: Table S5 — List of miRNAs differentially expressed in SALS fibroblasts compared to neurologically normal controls. [file nan0041-0201-sd5.docx]

**Supplementary Table 5:** microRNAs differentially expressed in SALS compared to control fibroblasts. All miRNAs were decreased in the SALS cases compared to controls. The reported known functions of each of the miRNAs are provided, as are any references to ALS. In addition, the miRNAs shown to have been previously differentially expressed following FUS knockdown in the neuroblastoma cell line SK-N-BE (Morlando et al, 2012) or in the spinal cord of ALS cases (Campos-Melo et al, 2013) are also highlighted.

| **miRNA** | **Functions described** | **Altered in FUS knockdown?** | **Altered in ALS spinal cord?** |
| --- | --- | --- | --- |
| hsa-miR-17 | HIF-1A represses the expression of miR-17 by down-regulating c-Myc expression. Overexpression of miR-17-92 cluster markedly inhibits hypoxia-induced apoptosis, whereas blocked miR-17-5p sensitizes the cells to hypoxia-induced apoptosis. | -0.61 | -0.53 |
| hsa-miR-20b | Regulates HIF-1A and VEGF and is regulated by HIF-1A so to keep tumor cells adapting to different oxygen concentrations. The VEGF expression in breast cancer cells is mediated by HIF-1 and STAT3 in a miR-20b-dependent manner. | -0.42 | -0.71 |
| hsa-miR-106a | Paralog to 17-92 cluster, oncogenic miRNA. | -0.69 | -0.51 |
| hsa-miR-107 | Up-regulated in hypoxia to prevent Endothelial progenitor cells differentiation by targeting HIF-1B. Also targets HIF-1A and negatively regulates VEGF signalling. Decreases hypoxia signalling by suppressing expression of HIF-1B. A toll-like receptor-regulated miRNA dysregulated in obesity and type II diabetes. | -2.32 | -1.15 |
| hsa-miR-125a-3p | Down regulated in human gastric cancer and non-small cell lung cancer. | -- | -0.72 |
| hsa-miR-142-5p | Decreased miR-142-3p/5p expression causes CD4+ T cell activation and B cell hyperstimulation in systemic lupus erythematosus. Down regulated in gastic cancer.  Increased in end stage SOD1 rat and mouse spinal cord (Koval et al, 2013) | -- | -0.85 |
| hsa-miR-149 | A methylation-sensitive miRNA, may play an important role as a tumour suppressor in colorectal cancer. Genetic variants in miR-149 reported but conflicting results for association with cancer risk. Also shown to be decreased in blood leukocytes from ALS patients (De Felice et al, 2012). | -0.54 | -0.83 |
| has-miR-155 | Oncogenic in multiple tumours. 147 validated targets from the literature, pathways inc apoptosis and angiogenesis. Represses HIF-1A expression, through direct binding to the 3’UTR of its mRNA. miR-155 induction contributes to an isoform-specific negative-feedback loop for the resolution of HIF-1A activity in cells exposed to prolonged hypoxia.  Showed increasing expression in monocytes and spinal cord microglia during disease progression in the SOD1 G93A transgenic mice (Butovsky et al, 2012)  Increased in the spinal cord of human ALS cases; inhibition of miR-155 increased survival of the SOD1 G93A mouse model by 38% (Koval et al, 2013) | +0.48 | -- |
| hsa-miR-219-1-3p | Enriched in human white matter and expressed in acutely isolated human oligodendrocytes | -- | -- |
| hsa-miR-324-3p | Dysregulation of the miR-324-3p/Prep pathway contributes to the development of fibrosis in progressive nephropathy. | -0.07 | -0.67 |
| hsa-miR-362-5p | None | -0.23 | -0.74 |
| hsa-miR-454 | None | -0.49 | -- |
| hsa-miR-484 | Can suppress translation of mitochondrial fission protein Fis1, and inhibit Fis1-mediated fission and apoptosis in cardiomyocytes and in the adrenocortical cancer cells. | -0.68 | -- |
| hsa-miR-589 | None | -- | -0.84 |
| hsa-miR-598 | Induces replicative senescence in human adipose tissue-derived mesenchymal stem cells via silent information regulator 1 | +0.82 | -0.79 |
| hsa-miR-616 | Induces androgen-independent growth of prostate cancer cells by suppressing expression of tissue factor pathway inhibitor TFPI-2. | -- | -- |
| hsa-miR-618 | None | +0.27 | -1.19 |
| hsa-miR-629 | Modulation of NBS1 gene expression (NBS1 involved in DNA repair) | -0.53 | -1.12 |
| hsa-miR-636 | None | +1.47 | -- |
